# Novel and selective inactivators of Triosephosphate isomerase with anti-trematode activity

**DOI:** 10.1038/s41598-020-59460-y

**Published:** 2020-02-13

**Authors:** Florencia Ferraro, Ileana Corvo, Lucia Bergalli, Andrea Ilarraz, Mauricio Cabrera, Jorge Gil, Brian M. Susuki, Conor R. Caffrey, David J. Timson, Xavier Robert, Christophe Guillon, Teresa Freire, Guzmán Álvarez

**Affiliations:** 1 Laboratorio de Moléculas Bioactivas, Universidad de la República, CENUR Litoral Norte, Paysandú, Uruguay; 2 Departamento de Química del Litoral, Universidad de la República, CENUR Litoral Norte, Paysandú, Uruguay; 3 Laboratorio de Reproducción Animal, Producción y Reproducción de Rumiantes, CENUR Litoral Norte-Facultad de Veterinaria, Universidad de la República, Paysandú, Uruguay; 4 Center for Discovery and Innovation in Parasitic Diseases, Skaggs School of Pharmacy and Pharmaceutical Sciences, University of California San Diego, La Jolla, CA USA; 5 School of Pharmacy and Biomolecular Sciences, The University of Brighton, Brighton, UK; 6 Equipe Rétrovirus et Biochimie Structurale, Université de Lyon, CNRS, MMSB, Lyon, France; 7 Laboratorio de Inmunomodulación y Desarrollo de Vacunas, Departamento de Inmunobiología, Facultad de Medicina, Universidad de República, Montevideo, Uruguay

**Keywords:** Drug discovery and development, Parasitic infection

## Abstract

Trematode infections such as schistosomiasis and fascioliasis cause significant morbidity in an estimated 250 million people worldwide and the associated agricultural losses are estimated at more than US$ 6 billion per year. Current chemotherapy is limited. Triosephosphate isomerase (TIM), an enzyme of the glycolytic pathway, has emerged as a useful drug target in many parasites, including *Fasciola hepatica* TIM (*Fh*TIM). We identified 21 novel compounds that selectively inhibit this enzyme. Using microscale thermophoresis we explored the interaction between target and compounds and identified a potent interaction between the sulfonyl-1,2,4-thiadiazole (compound **187**) and *Fh*TIM, which showed an IC_50_ of 5 µM and a K_d_ of 66 nM. In only 4 hours, this compound killed the juvenile form of *F. hepatica* with an IC_50_ of 3 µM, better than the reference drug triclabendazole (TCZ). Interestingly, we discovered *in vitro* inhibition of *Fh*TIM by TCZ, with an IC_50_ of 7 µM suggesting a previously uncharacterized role of *Fh*TIM in the mechanism of action of this drug. Compound **187** was also active against various developmental stages of *Schistosoma mansoni*. The low toxicity *in vitro* in different cell types and lack of acute toxicity in mice was demonstrated for this compound, as was demonstrated the efficacy of **187** *in vivo* in *F. hepatica* infected mice. Finally, we obtained the first crystal structure of *Fh*TIM at 1.9 Å resolution which allows us using docking to suggest a mechanism of interaction between compound **187** and TIM. In conclusion, we describe a promising drug candidate to control neglected trematode infections in human and animal health.

## Introduction

Trematode infections are a major cause of human disability and mortality in many developing countries, and remain one of the most important challenges for medicine in the 21st century^1–3^. An estimated 250 million people worldwide suffer these infections. Moreover, the *Fasciola* and *Schistosoma* parasites infect cattle, sheep and other animals of agricultural importance with estimated agricultural losses of more than US$ 6 billion per year^4^. In spite of their morbidity and economic impact, neither disease is of sufficient pharmaceutical industry interest for the development of new drugs, such that anthelmintic therapy relies precariously on just two drugs that were developed over 40 years ago: triclabendazole (TCZ) for fascioliasis and praziquantel (PZQ) for schistosomiasis^1,2^. The over-reliance on TCZ to treat sheep and, to a lesser extent, cattle, has resulted in selection for flukes resistant to TCZ. For PZQ, although clinically-relevant resistance has yet to emerge, resistance has been reported on occasion in the field and in various experimental settings^5–7^.

An important characteristic in the metabolism of trematode parasites is their dependence on glycolysis as an energy source for survival^8^. Thus, enzymes in the glycolytic pathway are attractive targets in the search for new small molecules chemotherapies^9,10^. One enzyme, triosephosphate isomerase (TIM; EC 5.3.1.1), is of particular interest as an anthelmintic target^8^. TIM catalyzes the isomerization of glyceraldehyde-3-phosphate and dihydroxyacetone phosphate in the fifth step of the glycolytic pathway^11^. It also has moonlighting functions, as it is found differently expressed in many types of cancer, it participates in the regulation of the cell cycle, function as an auto-antigen and in the evasion of the immune response, as a virulence factor in some organisms^12^. Structurally, most of the known TIMs are homodimers, each monomer consisting of eight parallel β-strands surrounded by eight α-helices that, together, form a typical “TIM barrel” fold. The interface between monomers occupies a significant portion of the molecular surface area of each monomer, approximately 1500 Å^2^ ^13–15^. Because TIM is active only in its dimeric form, small molecules that engage the interface may interfere with proper enzyme function^16^.

Encouraged by the fact that only 50% of the residues involved in the dimer interface are conserved between trematode TIMs and their human ortholog^17^, we aimed to identify molecules that specifically engage the parasite TIM interface. Accordingly, using 340 *in-house* compounds, we screened *in vitro* recombinant *Fh*TIM for low micromolar inactivators. With the best compounds, we performed selectivity assays (using mammalian TIMs) and preclinical studies (toxicology in mammalian cells and in mice). We evaluated their efficacy *in vivo* in mice infected with *F. hepatica* and perform X-Ray and docking studies to elucidate the mechanism of inhibition.

## Results

### Screening *Fh*TIM

**Figure 1:**
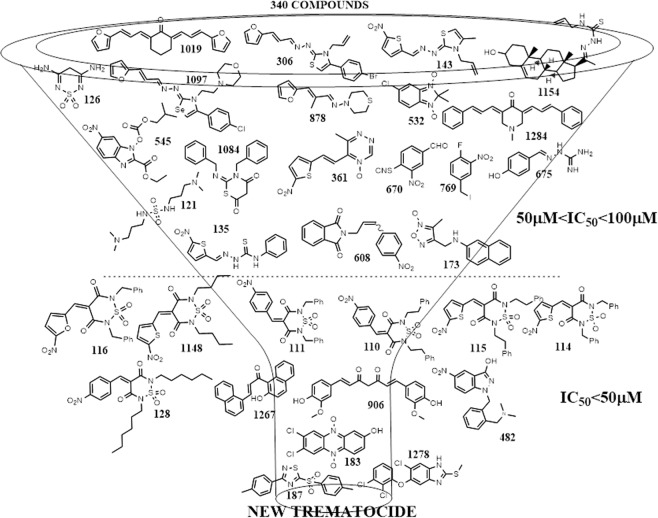
Inhibition cascade for a screen of 340 small molecules with recombinant *Fh*TIM. Structures of the best inhibitors are shown. Compounds are numbered according to Supplementary Table 1S. Three ranges of inhibitory activity were established: IC_50_ >100 µM (no activity, not shown), IC_50_ values between 50 and 100 µM (moderate activity) and IC_50_ <50 µM (good activity).

We hypothesized that a TIM dimer interface inactivator would be a successful strategy to identify molecules with selective antiparasitic activity^18–20^. Therefore, we selected 340 compounds from our *in-house* chemical collection and screened them against the isolated recombinant *Fh*TIM. In this work, the screened chemotypes are represented in Fig. 1 (benzofuroxanes, furanes and thiophenes, 4-substituted-1,2,6-thiadiazines, quinoxaline 1,4-dioxides, phenazine 5,9-dioxides, furoxanes, imidazole *N*-oxides, indazoles and others). Around 10% of the evaluated compounds were active with an IC_50_ <100 µM (concentration leading to at least 50% inhibition). Most of the families possess at least one active compound, except for the twenty evaluated quinoxalines from which no active molecules were found. Those families with a high percentage of active derivatives were the 4-substituted-1,2,6-thiadiazines, selenocompounds and phenazines, 60% (9/15), 100% (3/3) and 67% (2/3) active, respectively. We found 30–40% of inactivators among the thiadiazines and their precursors, and the curcuminoids family (Supplementary Table 1S). We established an IC_50_ <50 µM as the cut-off for good activity (Table 1). Only one active compound belongs to the thiadiazole chemotype (compound **187**, Fig. 1), which had the greatest inhibition of the molecular target (Table 1). Interestingly, **TCZ** (compound **1278** in the collection) inhibited *Fh*TIM with an IC_50_ of 7 µM (Table 1). In parallel, we performed MicroScale Thermophoresis (MST) with five representative molecules to evaluate their binding affinity. Among them, compound **187** showed the highest affinity for *Fh*TIM with the lowest IC_50_ (4.0 ± 0.2)_._

**Table 1:** IC_50_ values of the best TIM inactivators and experimental K_d_ values by MST.

Compound identifier	Structure	IC_50_ (µM)	K_d_ (µM)
110		7 ± 1	nd*
114		25 ± 8	664 ± 25
115		8 ± 2	120 ± 12
116		5 ± 1	nd
121		50 ± 9	nd
128		5 ± 1	120 ± 10
1148		15 ± 5	nd
135		50 ± 8	nd
310		79 ± 7	nd
144		60 ± 2	nd
1134		45 ± 4	nd
187		4.0 ± 0.2	0.07 ± 0.01
1278 (TCZ)		7 ± 1	1000
482		30 ± 3	nd
183		25 ± 3	nd
1149		50 ± 6	nd
906		35 ± 4	nd
1254		50 ± 5	nd
1267		35 ± 2	nd

*nd. not determined.

Then we performed an *in vitro* selectivity assay of *Fh*TIM as compared to human (*Hs*TIM) and rabbit TIM as a representative ruminant animal^21^. The best *Fh*TIM inhibitors, **110**, **115**, **128**, **187** and **TCZ**, were inactive (<20% inhibition) against *Hs*TIM and rabbit TIM at 100 µM.

### *In vitro* and *in vivo* anti-parasite activity of the compounds

**Figure 2:**
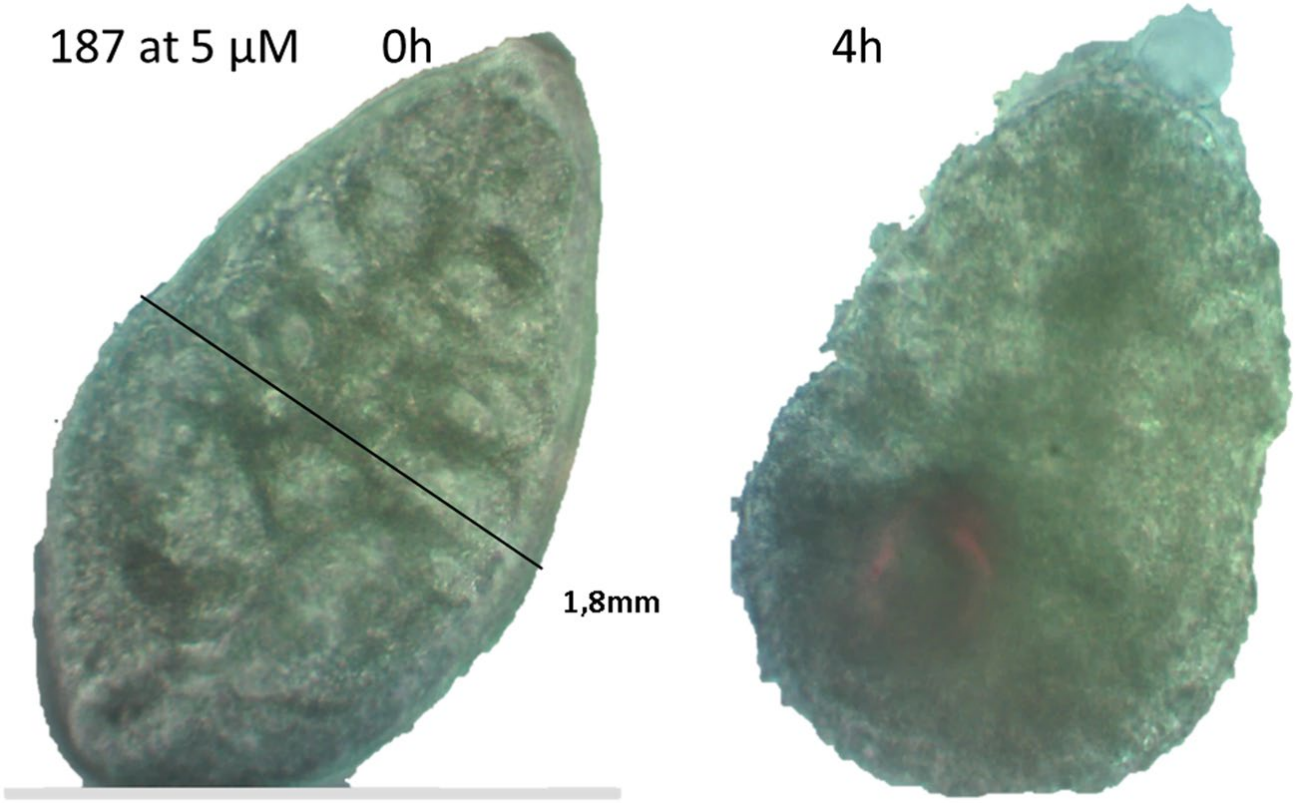
Optical microscopy (40×) photo of an NEJ from *F. hepatica* treated with compound **187** at 5 µM at 0 and 4 hours of incubation (supporting video).

As a proof of concept, we tested the best *Fh*TIM inactivators against *F. hepatica* and *S. mansoni* parasites. Also, we use compound **191** as a non-inhibitor which has a closely related structure to that of compound **187**. Some compounds were able to kill 100% of the newly excysted juvenile (NEJ) *F. hepatica* within 4 to 48 h (Table 2 and Fig. 2). Also, some of the compounds that were active against NEJ markedly affected *S. mansoni*, notably compound **187**, which, in the first 24 h, was lethal to somules, and decreased the motility of adults and the ability of their oral and ventral suckers to adhere to the floor of the culture dish well (Tables 2 and S2). In parallel, these compounds were assayed to characterize their non-specific toxicity against murine macrophages and bovine sperm. Both models are recommended by FDA as an *in vitro* prediction of toxicology^22^. We used fixed doses of 25 and 100 µM of compound to test against the mammalian cells: when the IC_50_ vs. the parasite was at least 10 times less than the IC_50_ vs. the mammalian cells, we considered these compounds as non-toxic (NT); if not, they were classified as toxic (T) (Table 2).

**Table 2:** The activity of the best compounds against juvenile forms of *F. hepatica* and *S. mansoni* somules.

Compound	*F. hepatica*		*S. mansoni* somules severity score*	Toxicity mammalian cells**
	IC_50_ (µM)	incubation time (h)		
906	7 ± 1	4	nd***	T
110	5 ± 2	30	0	T.
114	6.5 ± 0.6	4	4 (72 h)	T
115	7 ± 2	72	4 (72 h)	T
116	>50	72	Nd	nd
128	10 ± 1	12	Nd	T
1148	12 ± 2	4	0	nd
144	25 ± 5	72	4 (72 h)	NT
1134	50 ± 6	72	Nd	NT
187	3.0 ± 0.5	4	4 (24 h)^##^	NT
1293^#^	50	48	Nd	nd
1278 (TCZ)	>50	72	Nd	NT

*Severity score defined in Methods (*S. mansoni*: treatment of somules *in vitro* and adults *ex vivo*). **The selectivity index for the effect on the parasite vs. mammalian cells, NT represents an SI >10 when the toxicity was <50% at 100 µM vs. mammalian cells; ***nd, not determined; ^#^sulfonyl derivative of the TCZ active metabolite ^##^Supporting video.

Then we tested the biological activity of compound **187** against the adult form of *F. hepatica* and *S. mansoni*. This compound was selected as it was the most potent and least toxic of our compounds and is active against the juvenile stages of *F. hepatica*. In the case of *F. hepatica*, parasites were affected in the first 4 h at 10 µM (Fig. 2), ultimately leading to the death of all the parasites after 24 h. The *S. mansoni* parasite was also susceptible to compound **187**, showing decreased motility and loss of their ventral sucker adherence to the plate floor after 24 h at only 5 µM.

**Figure 3:**
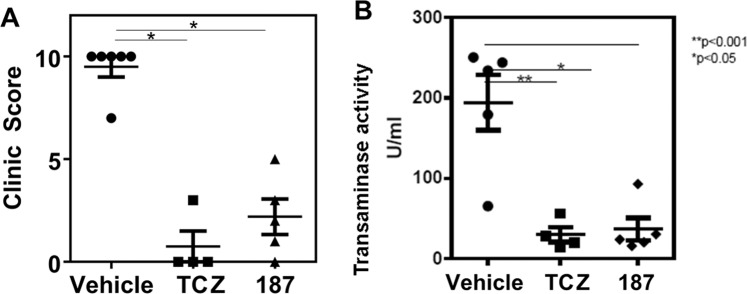
(**A**) Treatment of infected mice with one dose (100 mg/kg) of **187**, **TCZ** and as a control a lipidic emulsion (vehicle), (*p < 0.05). The “Clinic Score” is detailed in the methods section. (**B**) Transaminase profile at the end of the experiment.

Furthermore, the ability/potency of compound **187** to protect animals from *F. hepatica* infection was evaluated. To this end, BALB/c mice were infected with 10 *F. hepatica* metacercariae and one week later, compound **187** or **TCZ** were inoculated orogastrically. Compound **187**, together with **TCZ**, protected mice from the infection since they presented less severe clinical signs, including a decrease in liver necrosis, hemorrhage and splenomegaly (Fig. 3A). Then the transaminase profile was according to a healthy liver (Fig. 3B).

Finally, to get toxicological information about compound **187**, we performed acute oral toxicology (up-and-down experiment), which demonstrated an LD_50_ of ≥2000 mg/kg body weight in mice. An *in silico* exploration of the pharmacokinetic properties of compound **187** compared to the drug-likeness of other drugs gave parameters similar to those of the reference drug **TCZ** (Supplementary Fig. 2S).

### Crystal structure of *Fh*TIM and prediction of compounds binding sites

**Figure 4:**
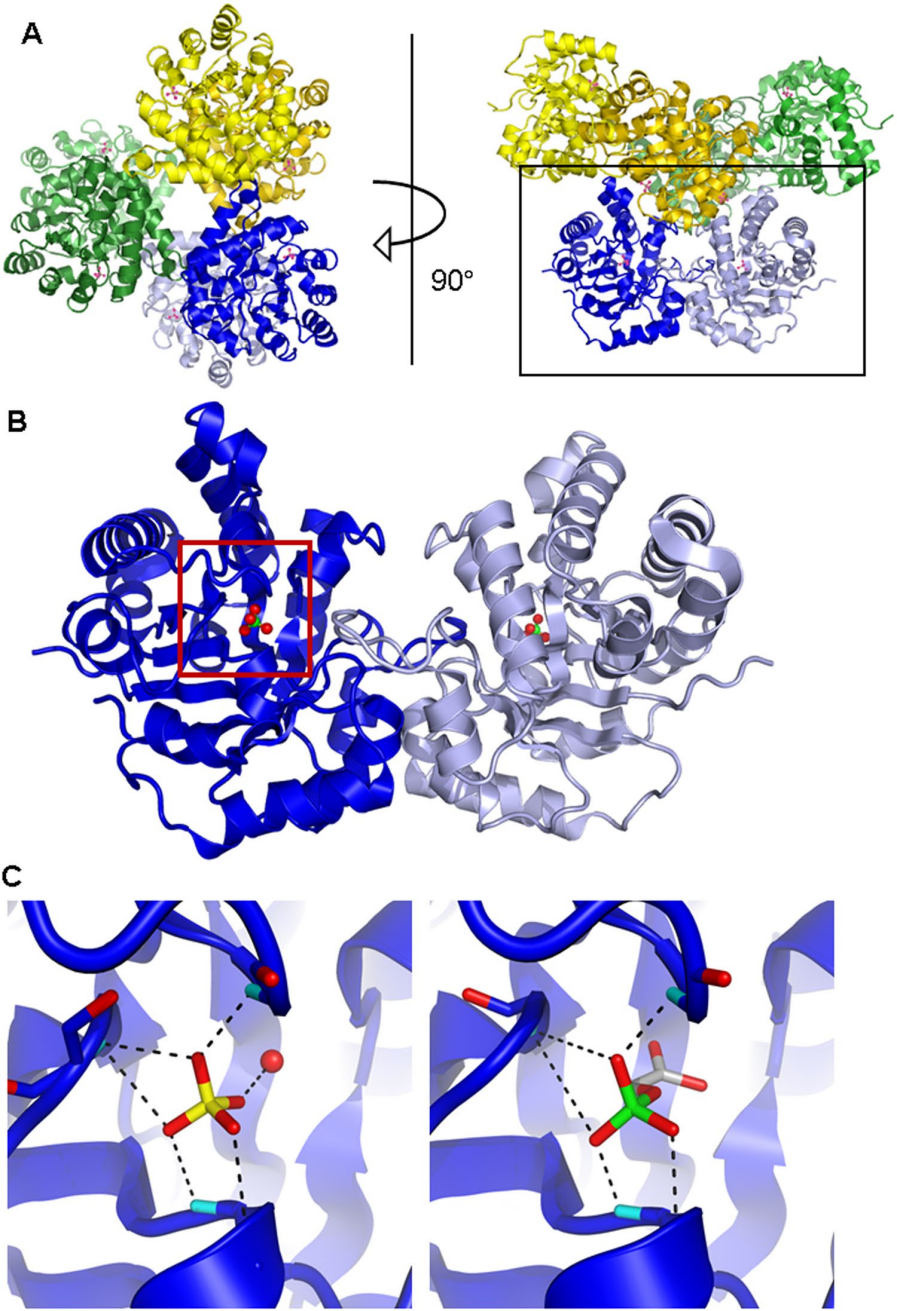
(**A**) Top (left) and side (right) view of the content of the asymmetric unit of *Fh*TIM crystal. Dimer A-D: blue tints; dimer B-C: green tints; dimer E-F: yellow tints, with one SO_4_^2−^ molecule (green and yellow) per protomer. Black rectangle highlights the dimer shown in B; (**B**) Close-up view of dimer A-D with the two SO_4_^2−^ molecules in the active site of each monomer (red and green). Red rectangle highlights the active site of *Fh*TIM shown in (**C**), left panel; (C) Close-up comparison of the active site of chain A of *Fh*TIM (left panel) with the active site of chain A of *Pf*TIM (PDB ID: 1LYX, right panel) with their liganded SO_4_^2−^ and phosphoglycolate (PGA), respectively. Dashed lines display the hydrogen bonds network of the ligand with neighboring residues/water molecules. The red sphere in the left panel corresponds to water molecule 465 of *Fh*TIM, chain A. Coloring scheme for the ligands’ atoms is: red = oxygen, yellow = phosphorus, green = sulfur, light gray = carbon, orange = chloride. The structure of *Fh*TIM is available in the PDB database (accession ID: 6R8H).

To elucidate the molecular basis of *Fh*TIM inhibition, we obtained the crystal structure of *Fh*TIM at 1.9 Å resolution. The asymmetric unit displays three *Fh*TIM dimers (Fig. 4A). Each protomer shows a typical (β/α)_8_ TIM-barrel fold. The R.M.S.D. between *Fh*TIM and human TIM (*Hs*TIM, PDB ID: 4POC)^23^ protomers is 4.5 Å on all Cα pairs, in spite of only a 68% sequence homology. The catalytic pocket of each protomer in the *Fh*TIM structure is occupied by a sulfate molecule (SO_4_^2−^), likely from the crystallization buffer. The β/α loop 6 (residues 168–180 of *Fh*TIM) is known to open and close when accommodating the substrate in triosephosphate isomerases. Here, the loops are in the close conformation in all of the protomers of *Fh*TIM (Fig. 4B). Interestingly, the SO_4_^2−^ ion is located in the same position as the phosphate group of the substrates (glyceraldehyde 3 P) in the *P. falciparum* (*Pf* TIM) or *S. aureus* TIM structures (PDB ID: 1LYX^15^ and 3UWU^24^, respectively) through similar interactions (Fig. 4C). Indeed, the three unbound oxygen atoms of the O_4_ tetrahedron in the triose phosphate moiety of *Pf* TIM interact with the main chain nitrogen of residues G175, S211, G236 and G237, as do 3 out of 4 oxygen atoms of the *Fh*TIM SO_4_^2−^. The remaining oxygen of the SO_4_^2−^ present in the *Fh*TIM structure (and spatially corresponding to the oxygen atom linking the phosphate to the sugar in the natural substrate) is coordinated by a water molecule roughly located at the position of the distal carbon of the substrate analog in *Pf* TIM (Fig. 4C). This suggests that the O_4_ tetrahedron drives the orientation of the substrate in the catalytic pocket.

Attempts to understand the mechanism of interaction between the inhibitors and TIM via crystallization did not yield ligand-bound structures. Thus, we used the crystal structure obtained for *Fh*TIM to perform docking studies with our compounds. For each compound, we performed 10 docking calculations including the 10 best poses, *i.e*., 100 positions selected per compound, which were then clustered and ranked. For each compound, the 10 best positions out of these resulting datasets are included in the supporting material. Docking was performed using four different strategies: “blind” docking on the whole surface of *Fh*TIM in the dimeric and the monomeric form, as well as targeted docking of only the active site with and without the flexibility of the residues in the active site pocket.

**Figure 5:**
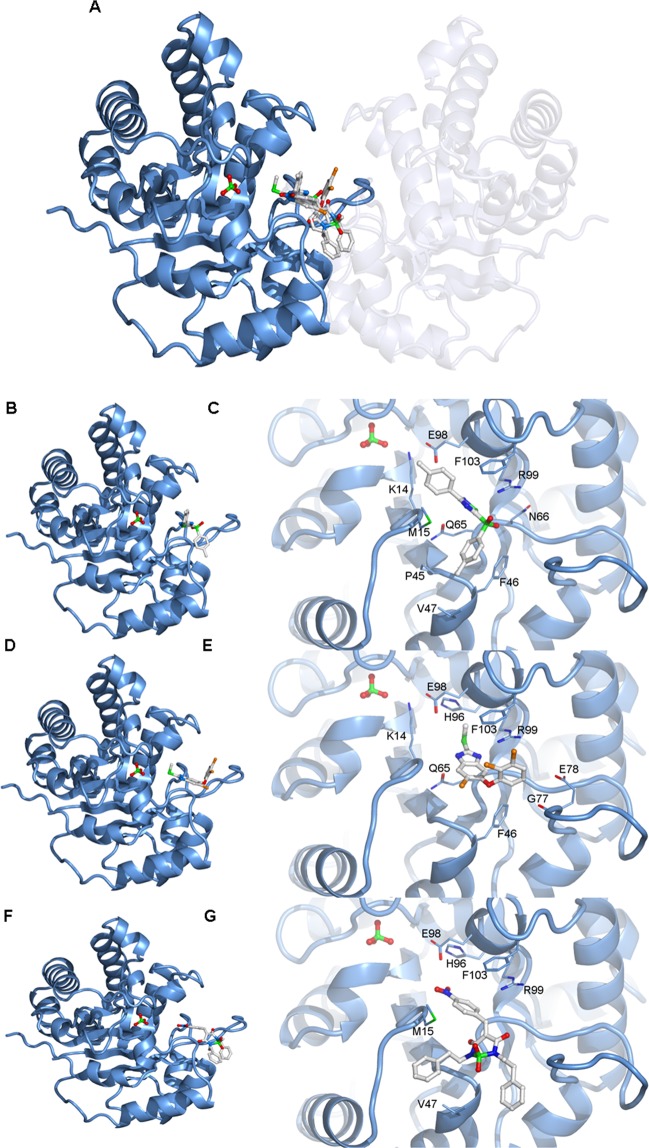
(**A**) Display of the best solutions for each compound after docking with compounds **110**, **187** and **TCZ** on the monomeric form of *Fh*TIM. The position of the second monomer in the dimer is displayed as a ghost to show the location of the docked compounds compared to the dimeric interface. Best solution after docking of compounds **110** (**B**,**C**), **187** (**D**,**E**) and **TCZ** (**F**,**G**) on the *Fh*TIM monomer. (**B,D,F**): Overall view of the position of the compounds. (**C,E,G**): close-up views of the position of the compounds with a display of the neighboring residues for which at least one atom lies within 4 Å of the ligand. These views are rotated 90° around the y-axis compared to panels B, D and F for the sake of clarity. The ligand coloring scheme is similar to Fig. 4C.

No preferential binding sites of the compounds could be identified on the surface of the dimer (Supplementary Material Fig. 1S). However, on the surface of the monomer, the compounds with the best IC_50_ in Table 1, including the anti-*F. hepatica* drug, TCZ, bind mostly to one region, which is directly involved in the dimerization (Fig. 5 and Supplementary Material Fig. 1S) with predicted affinities in the micromolar range. Given the size of the dimeric interface, these affinities would probably not be sufficient to disrupt a pre-formed dimer but the compounds might impair the dimerization of monomers or result in a modification of the dimer conformation. Noteworthy, compounds docked on the dimeric interface are predicted to bind close to crucial residues for TIM function. Indeed, the three representative compounds (**1278**, **110** and **187**) are located in close contact of K14 (less than 4 Å), and of these, **187** and **1278** (TCZ) are also predicted to interact with residue H96 (Fig. 5C,E,G). Both K14 and H96 are directly involved in the interaction of TIM with its natural substrate. Thus, the compounds, although not predicted to bind the active site, could inhibit interaction of TIM with its substrate. Anyhow, the docking experiments place the inactivators on a location of the dimer where they could have several effects on TIM function, which now need to be confirmed experimentally.

## Discussion

We discovered eleven small molecules belonging to four chemical classes (curcuminoids, thiadiazines, thiosemicarbazides and sulfones) that inhibit recombinant *Fh*TIM. This is the first report of inactivators on this enzyme. Interestingly, we found that **TCZ**, the reference drug in the treatment of fasciolosis, inhibits *Fh*TIM. **TCZ**’s mechanism of action is still debated. It has been suggested that **TCZ** destabilizes β-tubulin^25^, however, the direct evidence for interaction has not been demonstrated and the mechanism by which the parasites develop **TCZ** resistance does not appear to be the mutation of tubulin genes^25,26^. The hypothesis that **TCZ** interacts with tubulin is based on the better-known mode of action of other benzimidazoles for which tubulin interaction has been demonstrated in antiparasitic and anticancer applications^27^. The evidence for **TCZ**/tubulin interaction is weak and the structure of **TCZ**, in any case, is different from other anti-parasitic benzimidazoles^28^. Our findings open the possibility of exploring TIM as a biological target of this drug.

Solving the first crystal structure of *Fh*TIM provided insights both on the enzyme’s binding to natural substrates in the active site and the specific mechanism of action of our inactivators. Concerning the binding of substrate, the presence of a sulfate ion in the active site, and the fact that is orientated and coordinated as is the phosphate moiety of the substrate analog in *Pf* TIM^11^, suggests that the O_4_ tetrahedron is important for the correct orientation and coordination of the TIM substrate. Moreover, our finding of similar interactions between the SO_4_^2−^ and the phosphate moieties of the natural substrate in the active site could explain why sulfate ions inhibited *Trypanosoma* TIM^13,29^ *in vitro* and may facilitate orienting drug-design efforts focused on sulfated/phosphorylated molecules.

Docking suggests that the efficient *Fh*TIM inactivators (**187**, **110**, **116**, **128**), including **TCZ**, bind tightly at the enzyme’s dimerization interface (Fig. 5). As this dimerization is necessary for TIM function, our data suggest that our compounds could inhibit TIM by modifying or blocking the interface of the dimer. Inhibitors of protein:protein interactions have been sought for more than thirty years^19^ and although our compounds are small compared to the TIM interface, previous examples of small molecules inhibiting protein assembly have been described^30^. Moreover, in our case, as the catalytic site is at the dimeric interface, a disturbance of the topology of the interface, even if not leading to the disassembly of the dimer, could be sufficient to inhibit TIM function. Also, we describe using dynamic studies for *T. cruzi* TIM a particular movement in solution in the same region that allows strong interactions in small molecules like **187**^31^. Then, a conformational change near the active site, could affect the correct substrate interaction in the active site. In particular, according to our docking results, the region of the dimeric interface putatively targeted by **TCZ** and our compounds are also close to residues K14 and H96, which are directly involved in the interaction of TIM with its natural substrate. Thus, **187** and **TCZ** may inhibit the binding of TIM to its substrate by blocking the catalytic loop in the closed, unfavorable conformation. Given the vicinity of the two zones, one cannot exclude that these two effects are involved in the inhibition by compounds **187** and **TCZ**. Such a dual effect would be very interesting from a pharmaceutical point of view, as the parasites would need to accumulate mutations against these two effects in order to become resistant to the treatment. Our docking data are *in silico* predictions, but they warrant further experiments to seek for a functional confirmation of the molecular mechanisms of inhibition by compounds **187** and **TCZ**.

The best *Fh*TIM inactivators are lethal to both juvenile and adult forms of *F. hepatica*, essentially immobilizing both developmental forms at 10 µM after 4 h, a significant improvement over the activity of **TCZ** against both forms (the effect of **TCZ** was at 48 h at more than 50 µM). Because TIMs are conserved proteins, including among trematodes^8,31^, we demonstrated that the best *Fh*TIM inactivator compounds were also active against somules and adults of the *S. mansoni* bloodfluke. Of these, **144**, **1134** and **187**, were non-toxic to mammalian cells both herein, and previously at 100 µM against murine macrophages and human peripheral blood mononuclear cells^32^. They were also more active against both parasites than the reference drug TCZ and its active metabolite **1293** (6-chloro-5-(2,3-dichlorophenoxy)-2-(methylsulfonyl)-1*H*-benzo[d]imidazole). The biological activity of **187** was observed immediately and developed faster than the other compounds. By contrast, it took several hours for **TCZ** to kill *Fasciola* NEJ *in vitro*. In addition, **187** displayed low toxicity in the acute oral toxicity assay. Moreover, its pharmacokinetic profile is comparable with commercially available drugs. This compound is also a moderate inhibitor of thioredoxin glutathione reductase (an essential core enzyme for redox homeostasis in flatworm parasites) and showed effect on NEJ and *Echinococcus granulosus* protoscolex at 48 h and a fixed dose of 20 µM^32^. Also compound **187** protect mice infected with *F. hepatica* like the reference drug. We identified compound **187** as a lead candidate and obtained *Fh*TIM structure at 1.9 Å resolution crystals to understand the compound’s ligand-binding characteristics. Compound **187** is the first, selective, anthelmintic TIM inactivator reported with *in vivo* efficacy. Altogether, our findings suggest that compound **187** is a good drug candidate for the treatment of flatworm infections. As these zoonotic parasites affect human and animal health, our compound **187** seems a good lead to developing a new class of “all-in-one” drugs with broad anti-parasitic potential.

## Methods

### Chemicals

The studied compounds were selected from our chemo library (LIDENSA) using the following criteria: (i) agents belonging to *T. cruzi* TIM inhibitors, or/and (ii) symmetrical and benzo-containing agents, structurally related to previously described TIM inhibitors. The selected compounds belong to fourteen chemotypes: 1-thiazoles, 2-thiadiazoles, 3-quinoxalines, 4-thiosemicarbazides, 5-steroids, 6-thiadiazines and precursors, 7-selenocompounds, 8-hydrazines, 9-curcuminoids, 10-indazoles, 11-imidazoles, 12-benzo-furoxanes, 13-triazines, 14-phenazine and 40 molecules with diverse structures not clustered in any family. The purity of the compound was checked only with the active molecules, reaching more than 98% by HPLC-MS. The synthetic procedures for the selected active compounds were described previously compound **187**^33^, thiadiazine compounds^34^, compound **482**^35^. Compound **1278** is **TCZ**, which is a commercially available anti-fascioliasis drug (batch PS059349, 98.9% pure from Andres Pintaluba S.A.)

### Microscale thermophoresis

MST experiments were performed according to the NanoTemper technologies protocol in a Monolith NT.115 (red/blue) instrument (NanoTemper Technologies, München, Germany^36^). With this technique, the diffusion behavior of a labeled protein is measured when an infrared light excites the movement of the protein in capillaries. This behavior is the combination of two effects: the fast, local environment-dependent responses of the fluorophore to the temperature jump and the slower diffusive thermophoresis fluorescence changes. It will be modified when the protein is complexed with increasing amounts of unlabeled partners, leading to titration curves that can be fit for K_d_ estimation^36^. In practice, *Fh*TIM was labeled with the Monolith His-Tag Labeling Kit RED-tris-NTA (NanoTemper Technologies, München, Germany), as described by the manufacturer. The experiments were performed using 20% and 40% MST power and between 20–80% LED power at 24 °C. The MST traces were recorded using the standard parameters: 5 s MST power off, 30 s MST power on, and 5 s MST power off. The compounds were used at high concentrations (around 5 mM) in the bindings check assay with DMSO at 5% v/v. If the binding check was positive, then the affinity determination was performed with the same experimental settings, with serial 2x dilutions of the compound of interest.

### Inhibition of Triosephosphate Isomerase

Expression and purification of proteins: *Fh*TIM, RabbitTIM and *Hs*TIM were expressed in *Escherichia coli* and purified as described in the literature^31,37^. After purification, the enzyme, dissolved in 100 mM triethanolamine, 10 mM EDTA and 1 mM dithiothreitol (pH 8), was precipitated with ammonium sulfate (75% saturation) and stored at 4 °C. Before use, extensive dialysis against 100 mM triethanolamine/10 mM EDTA (pH 7.4) was performed. Protein concentration was determined by absorbance at 280 nm for *Fh*TIM (ε = 33,460 M^−1^cm^−1^) and for RabbitTIM and *Hs*TIM (ε = 33,460 M^−1^cm^−1^). Enzymatic activity was determined following the conversion of glyceraldehydes-3-phosphate into dihydroxyacetone phosphate in a coupled enzyme assay. The decrease in absorbance at 340 nm was followed in a multiplate reader VarioskanTM Flash Multimode Reader (Thermo ScientificTM, Waltham, MA, USA) at 38 °C. The reaction mixture (1 mL, pH 7.4) contained 100 mM triethanolamine, 10 mM EDTA, 0.2 mM NADH, 1 mM glyceraldehydes-3-phosphate and 0.9 units of α-glycerol phosphate dehydrogenase. The reaction was initiated by the addition of 5 ng/mL of the TIM of interest. For the inhibition studies, TIM was incubated at a concentration of 5 mg/mL in a buffer containing 100 mM triethanolamine, 10 mM EDTA, pH 7.4 and 10% of DMSO at 37 °C for 1 h. The mixture also contained the compounds, dissolved in DMSO, at the indicated concentrations. After 1 h, 10 µL were withdrawn and added to a final volume of 100 µL of the reaction mixture for the activity assay. The inhibition assay was performed in a 96-well microplate. None of the molecules tested here affected the activity of α-glycerol phosphate dehydrogenase, the enzyme used in the coupled assay. The IC_50_ value was taken as the concentration of drug needed to reduce the enzymatic activity to 50% by analysis using OriginLab8.5® sigmoidal regression (% of enzymatic activity vs. the logarithm of the compound concentration). The experiments were performed in triplicate in two independent experiments.

### *Fh*TIM crystallization

*Fh*TIM was resuspended at 30 mg/mL in 100 mM triethanolamine/10 mM EDTA (pH 7.4) and stored at −80 °C until use. Protein and crystallization conditions were equilibrated at 19 °C for two hours before preparing the drops. The screening was performed in 96-wells plates using a Mosquito Nanopipetter (TTP Labtech) and commercial screens (Hampton Research, Qiagen) with the sitting drop method, at 19 °C in a Rockimager crystal farm (Formulatrix). Hits grew within 2 weeks in condition B2 of the PEGs II screen (Qiagen). Bigger crystals were grown using the hanging drop method by mixing 1 to 2 µL of protein solution with an equal amount of crystallization condition (0.1 M MES pH 6.5, 0.01 M ZnSO_4_, 50% PEG550mme). Crystals appeared within two weeks and were directly snap-frozen in liquid nitrogen before being submitted to X-ray diffraction.

### X-ray data collection and structure determination

X-ray data were collected at 1.9 Å on beamline ID23-1 from European Synchrotron Radiation Facility, Grenoble, France at 100 K using wavelength λ = 0.97242 Å. Data were processed in space group P3_1_ with cell dimensions a = b = 87.4 Å c = 186.6 Å, α = β = 90° and γ = 120°. Indexation and scaling were performed using XDS and XSCALE programs^38^_._ Molecular replacement was performed with the Phaser software^39^ and using the A chain of Chicken TIM (69% sequence homology, PDB: 4P61)^40^ as a search model with 6 molecules in the asymmetric unit. Initial refinement was performed with Phenix^41^ using twin law -*h-k, k, -l*. Water molecules were added during Phenix refinement. After manual curation of the structure (manual placement of the β/α loop 6 (residues 168–180), manual addition of 6 molecules of SO_4_, tidying up water molecules) using WinCoot^42^, further refinements were performed using Refmac 5.8.0238 from the CCP4 suite^43^. The structure was refined to a final R_work_ of 19.6% and R_free_ of 22.4%, respectively. Statistics of the X-ray data are shown in Table 3. Geometry analysis using Rampage^44^ showed 96% of the residues in preferred regions, 3.6% in allowed regions, and 6 residues (0.4%) as Ramachandran outliers (residues 102 and 156 from chain B, E and F).

**Table 3:** Summary of X-ray data collection and refinement statistics. Values in parentheses are for the highest-resolution shell.

Data collection	*Fh*TIM	Refinement	*Fh*TIM
Space group	P3_1_	Resolution range (Å)	20-1.9
Unit cell parameters			
*a, b, c* (Å)	87.4, 87.4, 186.6	Number of unique reflections	107970
α, β, γ (°)	90, 90, 120		
Resolution range (Å)	20-1.9 (1.95-1.9)	R_work_ (%)	19.6
R_sym_ (%)	14.4 (152.5)	R_free_ (%)	22.4
I/σI	5.11 (0.76)	Number of proteins atoms	11568
Completeness (%)	98.7 (92.9)	Number of water/SO4	1116/6
Redundancy	5.15	Mean B-factor (Å^2^)	18.13
		**Coordinate deviations**	
		RMSD bond lengths (Å)	0.01
		RMSD angles (°)	1.9
		**PDB ID**	**6R8H**

### Docking

The present crystal structure of *Fh*TIM was used as a target in the subsequent docking studies. Compounds were modeled using their SMILES codes from the Chemoffice software. Then, both target protein and ligands were prepared using AutoDockTools v1.5.6^45^: the polar hydrogen atoms were added, the non-polar hydrogens were merged, and the Gasteiger partial atomic charges were calculated. Finally, all the possible rotatable bonds were assigned for each compound. Four distinct docking experiments were then carried out with the program AutoDock Vina v1.1.2^46^: a search on the entire surface of *Fh*TIM in its dimeric and monomeric forms as well as a targeted docking in the active site only, with and without flexibility of the side chains of the residues defining the binding pocket. Compounds were treated as fully flexible in each experiment. The search grid was defined accordingly in order to encompass the considered areas. A visual examination of the resulting poses was performed using PyMOL (Schrödinger, Delano Scientific, LLC, New York, NY, USA).

### Cell culture

J774.1 murine macrophage cells (ATCC, USA) were grown in DMEM culture milieu containing 4 mM glutamine and supplemented with 10% FCS^20^. The cells were seeded in a 96-well plate (5×10^4^ cells in 200 µL culture medium) and incubated at 37 °C in a 5% CO_2_ atmosphere for 48 h, to allow cell adhesion prior to drug testing. Afterward, cells were exposed for 48 h to the compounds (25–400 µM) or the vehicle for control (0.4% DMSO), and additional controls (cells in medium) were used in each test. Cell viability was then assessed by measuring the mitochondria-dependent reduction of MTT (3-(4,5-dimethylthiazol-2-yl)-2,5-diphenyltetrazolium bromide) to formazan. For this purpose, MTT in sterile PBS (0.2% glucose), pH 7.4, was added to the macrophages to achieve a final concentration of 0.1 mg/mL, and the cells were incubated at 37 °C for 3 h. After removing the medium, formazan crystals were dissolved in 180 µL of DMSO and 20 µL of MTT buffer (0.1 M glycine, 0.1 M NaCl, 0.5 mM EDTA, pH 10.5), and the absorbance at 560 nm was measured. The IC_50_ was defined as the drug concentration at which 50% of the cells were viable, relative to the control (no drug added), and was determined by analysis using OriginLab8.5® sigmoidal regression (% of viable cells vs. the logarithm of the compound concentration). Tests were performed in triplicate.

### Cytotoxicity assay on bovine spermatozoa

Semen samples were obtained from a healthy fertile Hereford bull and kept frozen in 0.5 mL straws (extended in Andromed, Minitube, Germany^47^) under liquid nitrogen until use. The semen used belonged to a single freezing batch that was obtained during a regular collection schedule with an artificial vagina. Samples from three straws were thawed and a sperm pool was prepared in PBS at a concentration of 40 million spermatozoa per mL, then 50 μL of this sperm suspension was carefully mixed with 50 μL of compounds diluted to 100, 50, 25, 12.5 and 6.25 μM or with 1% DMSO in control experiments. Each condition was assayed by duplicate in 96-well plates and controls were assayed by triplicate. Plates were incubated at 37 °C for 1 h with moderate shaking. The motility analysis was carried out using a CASA (Computer Assisted Semen Analyzer) system Androvision (Minitube, Tiefenbach, Germany) with an Olympus BX 41 microscope (Olympus, Japan) equipped with a warm-stage at 37 °C. Each sample (10 μL) was placed onto a Makler Counting Chamber (depth 10 μm, Sefi-Medical Instruments, Israel) and the following parameters were evaluated: percentage of total motile spermatozoa (motility >5 μm/s) and velocity curved line (VCL, >24 μm/s). At least 400 spermatozoa were analyzed from each sample from at least four microscope fields.

### *In vitro* NEJ treatment

*F. hepatica* metacercariae were acquired from DILAVE, MGAP, Uruguay. NEJ were obtained by *in vitro* excystement as previously described with minor modifications^47^. Briefly, metacercariae were incubated with 1% sodium hypochlorite for 5 min at room temperature to remove the outer cyst wall and then washed exhaustively with PBS 200 U/mL Penicillin G sulfate, 200 mg/mL streptomycin sulfate, 500 ng/mL amphotericin B, 10 mM HEPES, counted and divided into groups of around 20 parasites that were transferred to 12 wells tissue culture plates. Parasites were maintained at 37 °C, 5% CO_2_ in modified Basch’s medium^47^. At day 1, compounds were added at the indicated concentrations and 0.5% DMSO was added to control groups; each condition was tested in duplicate. NEJ behavior was monitored under a light microscope (Olympus BX41), every day each well was recorded for a minute in order to assess parasite motility and registered using the following score: 3- normally active; 2- reduced activity (sporadic movement); 1- immotile (dead)^48^.

### *Ex-vivo* in the adult form of *F. hepatica*

*F. hepatica* were collected from natural infections of cattle obtained from Casablanca slaughterhouse in Paysandú, Uruguay^49^. During slaughter (500 animals observed) the livers that showed signs of fascioliasis (thickened canaliculi) were dissected and adult flukes were recovered and kept in PBS at 37 °C until used (no more than 8 h). After flukes emptied their gut content (i.e. the gut was not dark anymore) they were transferred to 6 well plates (one fluke per well) with 2 mL of RPMI 1640 media with 200 U/mL Penicillin G sulfate, 200 mg/mL streptomycin sulfate, 500 ng/mL amphotericin B and 10 mM glucose at 37 °C. Compounds were added at a concentration of 25 µM. The antiparasitic efficacy was expressed as the percentage reduction of the number of flukes in the treated group compared with the control group.

### *S. mansoni*: treatment of somules *in vitro* and adults *ex vivo*

The NMRI isolate of *S. mansoni* was maintained by passage through *Biomphalaria glabrata* snails and 3–5 week-old, female Golden Syrian hamsters (Charles River, San Diego, CA) as intermediate and definite hosts, respectively. A dose of 600 infective larvae (cercariae) was used to infect hamsters. The acquisition, preparation and *in vitro* maintenance of *S. mansoni* post-infective larvae (schistosomula or somules) and adults have been already described^5,50,51^. Vertebrate animal maintenance and handling at the University of California San Diego Animal Care Facility were in accordance with protocols approved by the university’s Institutional Animal Care and Use Committee (IACUC).

Phenotypic screens with somules were performed as described^5,50,51^. Initially, 100 μL Basch medium^52^, 100 U/ml penicillin, 100 mg/ml streptomycin, 4% heat-inactivated FBS (Corning Mediatech) and 1 μl compound in DMSO were added to clear, 96-well round-bottomed plates (Costar cat.# 3367). Then, 100 μL of the same medium containing 40–50 somules was added to mix the compound with somules (final concentration of DMSO was 0.5%). Assay plates were placed into plastic boxes humidified with wet tissue and then incubated at 37 °C in a 5% CO_2_ environment. Somules phenotypes were recorded every 24 h up to 72 h using an inverted microscope (Zeiss Axiovert 40 C).

Phenotypic screens with 42-day-old, adult parasites were performed in 24-well plates (Costar cat.# 3526) containing the above medium and approximately five adult males and two females per well. Compound was added in a volume of up to 1 μL DMSO at a concentration of 5 µM (0.05% DMSO final). Assay plates were incubated at 37 °C in a 5% CO_2_ environment. Parasite phenotypes were recorded at 4, 8 and 24 h using a Zeiss Axiovert 40 C.

Schistosome phenotypic responses were recorded employing a series of ‘descriptors,’ such as rounding, degeneration, overactivity, loss of translucency and changes in motility, as described previously^5,50,51,53^. To allow for comparisons of compound activity, each descriptor was awarded a value of 1 and these were added up to a maximum ‘severity score’ of 4. Evidence of degeneracy or death was awarded the maximum score of 4. Scores were averaged across duplicate wells for each compound (see details in the supporting information).

### Acute oral toxicity

The *in vivo* 50% lethal dose (LD_50_) was determined according to the guidelines of the Organization for Economic Cooperation and Development (OECD)^54,55^. All procedures involving animals were approved by the Universidad de la República’s Committee on Animal Research (CHEA Protocol ID 707). Briefly, healthy young adult male BALB/c mice (30 days old, 25 to 30 g) were used in this study. Initially, Compound **187** dissolved in vehicle was administered at 2,000 mg/kg, by orogastric cannula, to one animal. The animal was fasted, maintained, and observed for 14 days according to the OECD guidelines. If the mouse survived for the first 48 h, another animal received the same dose. If this was repeated, a third animal was dosed with 2,000 mg/kg and also observed for 14 days. The experiment ends at 14 days post-administration, if there are no signs of toxicity, the (LD_50_) is the administered dose, if not, the software AOT AOT425 Stat program recommendation was followed. For the vehicle preparation^56^ compound **187** was disposed in a mixture, composed of a surfactant (10%), containing Eumulgin HRE 40 (polyoxyl-40hydrogenated castor oil), sodium oleate, and soya phosphatidylcholine (8:6:3), and an oil phase (10%) containing cholesterol and phosphate buffer (pH 7.4) (80%). Compound **187** was pulverized in a mortar with cholesterol, Eumulgin HRE 40, and phosphatidylcholine, then the mixture was dissolved in chloroform and the solvent was evaporated under vacuum to dryness. In parallel, sodium oleate was dissolved in phosphate buffer and left in an orbital shaker for 12 h at room temperature. The former was then added to the evaporated residue, and the mixture was homogenized and placed in an ultrasonic bath at full power for 30 min. The sample was kept at room temperature until use.

### Mouse infection with *F. hepatica*

Six- to eight-week-old female BALB/c mice were obtained from DILAVE Laboratories^57^ (Uruguay). Animals were kept in the animal house (URBE, Facultad de Medicina, UdelaR, Uruguay) with water and food supplied ad libitum. Mouse handling and experiments were carried out in accordance with strict guidelines from the National Committee on Animal Research (CNEA, Uruguay). All procedures involving animals were approved by the Universidad de la República’s Committee on Animal Research (CHEA Protocol Number: 070153-000180-16). BALB/c mice were orally infected with 10 *F. hepatica* metacercariae per animal. After 1 week post-infection (wpi), mice were intragastrically inoculated with compound **187** (100 mg/kg) or **TCZ** (100 mg/kg). At 3 wpi mice were bled and sacrificed. Peritoneal exudate cells, spleens, and livers were removed and analyzed. In order to evaluate the severity of the infection, a disease severity score was developed (Table 3S Supporting Information)^57^, which was applied in blinded experiments. Alanine aminotransferase (ALT) activity in sera was determined using a commercial kit (Spinreact, Spain) according to the manufacturers’ instructions. PECs from infected and non-infected mice were washed twice with PBS containing 2% FBS and 0.1% sodium azide.

### Ethical approval

The study was approved by the Universidad de la República’s Committee on Animal Research (CHEA Protocol Number: 070153-000180-16 and ID 707) Review Boards. Under the rules of Comisión Nacional de Experimentación Animal: (CNEA, http://www.cnea.gub.uy/).

### *In silico* pharmacokinetic parameters

The predictions were obtained from the online free software SwissADME (http://www.swissadme.ch): a free web tool to evaluate pharmacokinetics, drug-likeness and medicinal chemistry friendliness of small molecules^58^. The smiles codes were provided for the software to calculate and the parameters.

### Statistical analysis

All statistical tests were performed using Microsoft Excel. Student’s t-test (two-tailed distribution) was used to calculate *P* values. The number of experiments and samples was described in the figure legends. All data obtained in more than three experiments or samples show the mean ± s.d. Nonlinear least-squares fitting was performed using KaleidaGraph Ver. 4.5 to evaluate inhibition activity of compounds (IC_50_).

## Supplementary information

Supporting information.

187 at 10uM in Smansoni adults.

control of Smansoni adults.

187 5 uM _12h nej Fhepatica.

control 12h nej Fhepatica.
