# Supplementary material for: Novel and selective inactivators of Triosephosphate isomerase with anti-trematode activity
Source: Sci Rep. 2020 Feb 13;10:2587. doi: 10.1038/s41598-020-59460-y (PMC7018972; doi:10.1038/s41598-020-59460-y)
Supplement: Supplementary file 1 — Supporting information. [file 41598_2020_59460_MOESM1_ESM.pdf]

Novel and selective inactivators of Triosephosphate isomerase with anti-trematode activity.

Florencia Ferraro[1#], Ileana Corvo[1#], Lucia Bergalli[2], Andrea Ilarraz[1], Mauricio Cabrera[1], Jorge Gil[3], Brian M. Susuki[4], Conor R. Caffrey[4], David J. Timson[5], Xavier Robert[6], Christophe Guillon[6], Teresa Freire[7], Guzmán Álvarez[1]*

Table 1S. the whole results from the screening with 340 compound in *Fh*TIM		
**ID_Mol**	**STRUCTURE**	**IC50**
**THIAZOLES**		
258	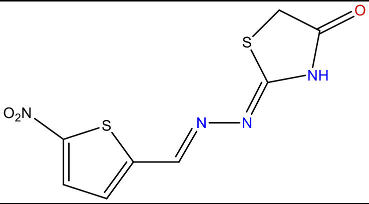	>100
273	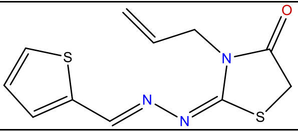	>100
262	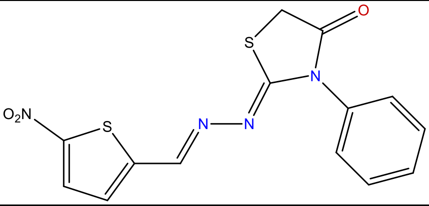	>100
260	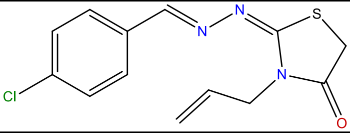	>100
264	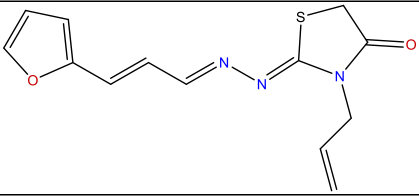	>100
268	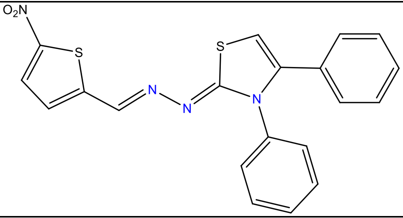	>100
147	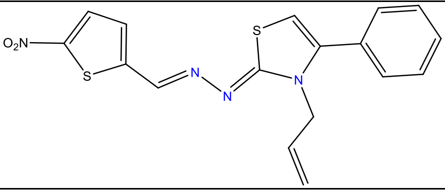	>100

791	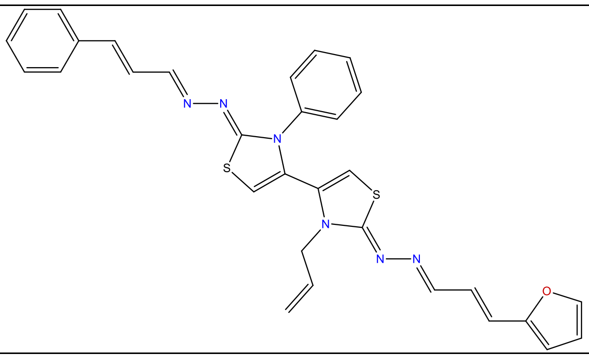	>100
1093	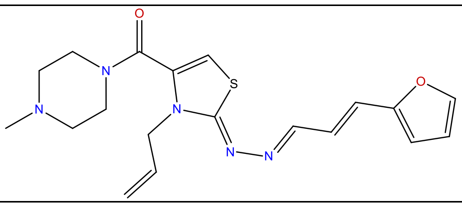	>100
276	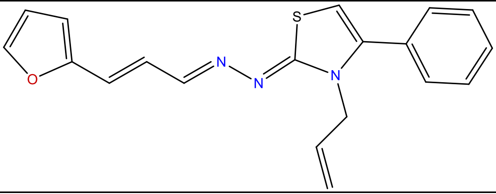	>100
259	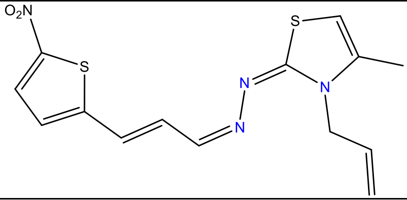	>100
272	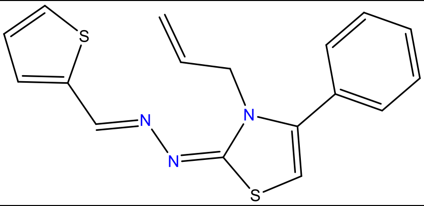	>100
267	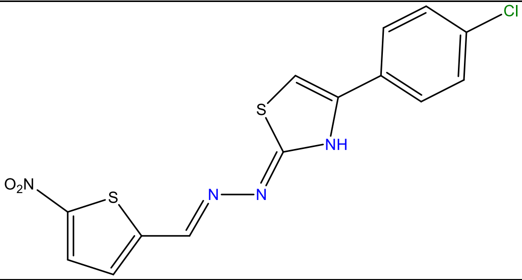	>100
143	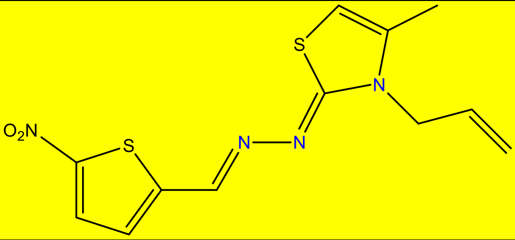	50-100
274	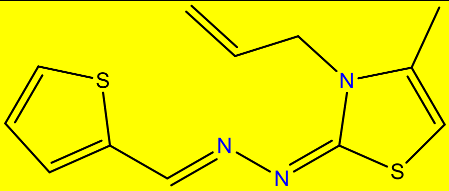	50-100

265	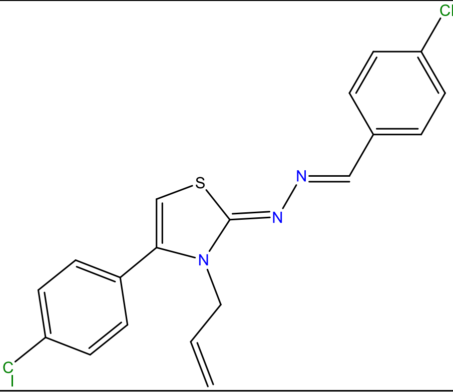	>100
314	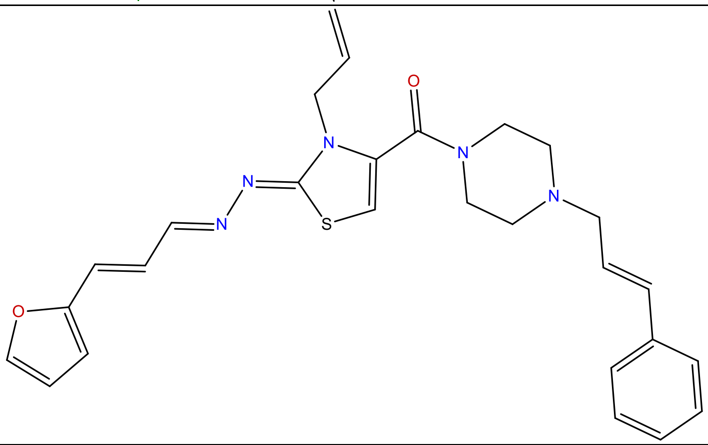	>100
1099	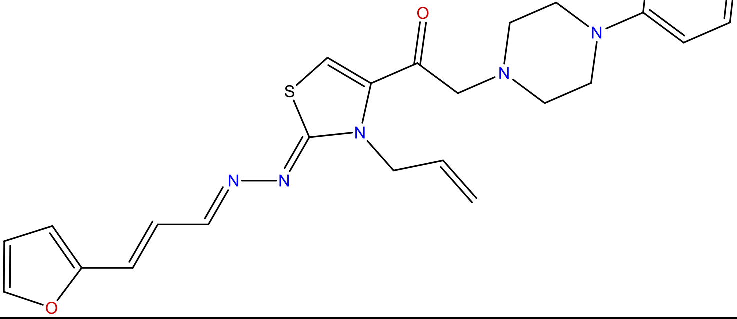	>100
872	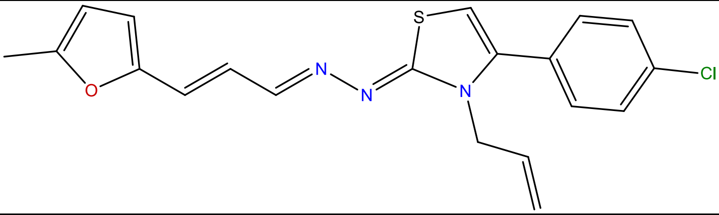	>100
1103	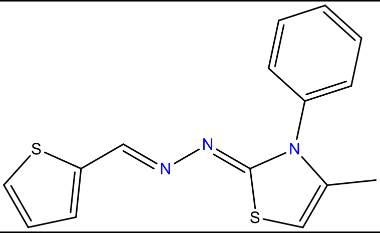	>100
810	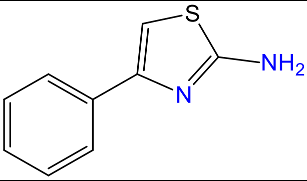	>100
1098	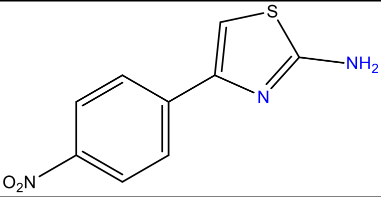	>100

288	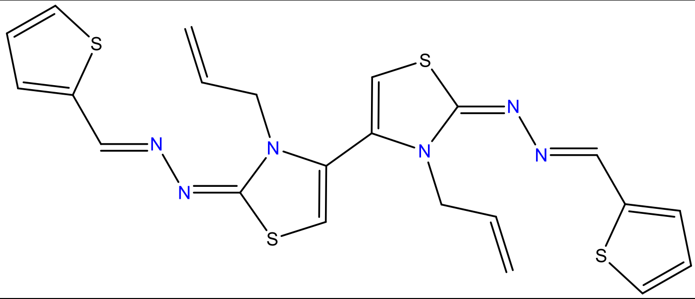	>100
266	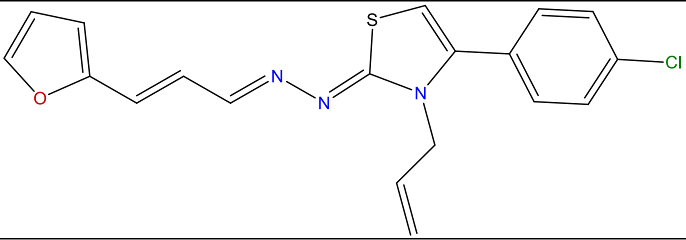	>100
132	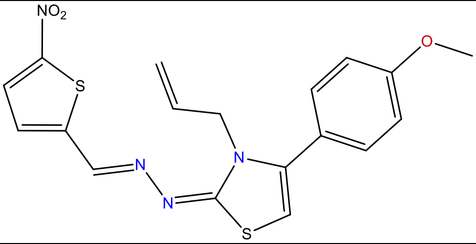	>100
145	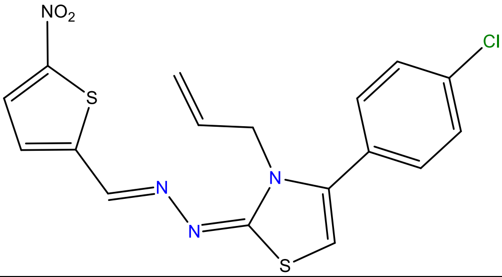	>100
146	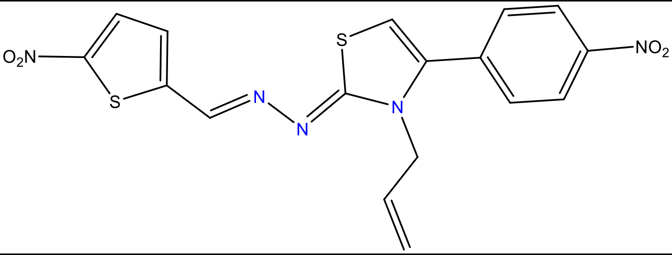	>100
1100	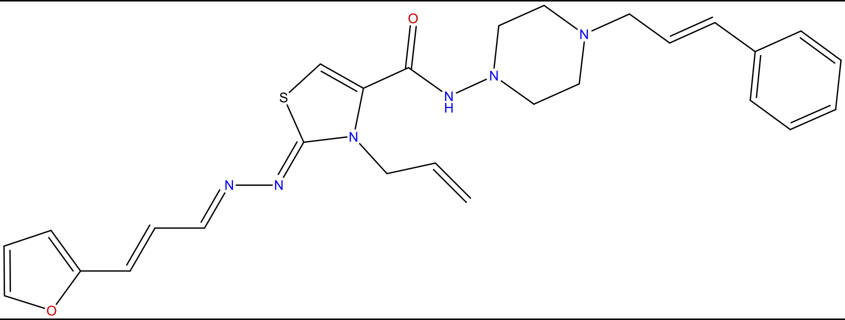	>100
129	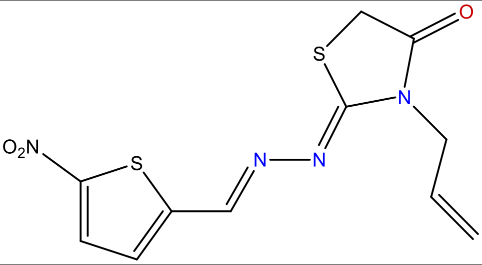	>100

1115	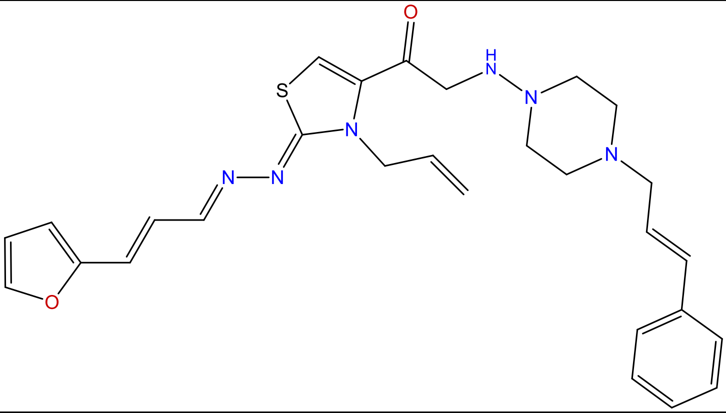	>100
785	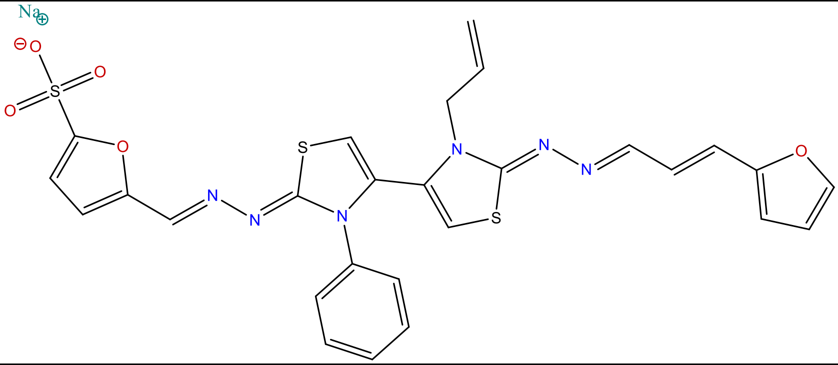	>100
781	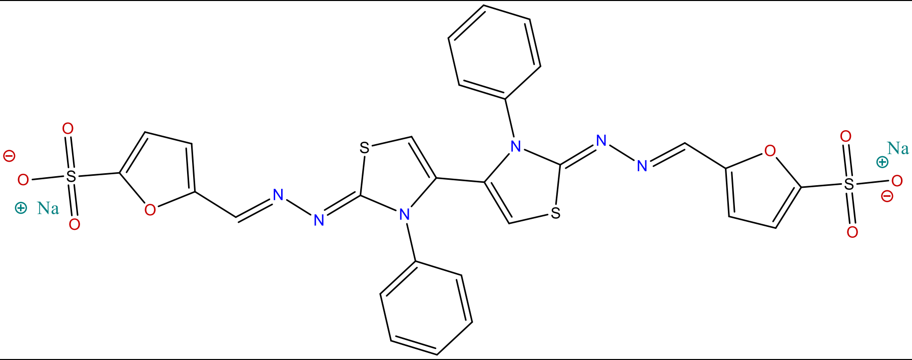	>100
783	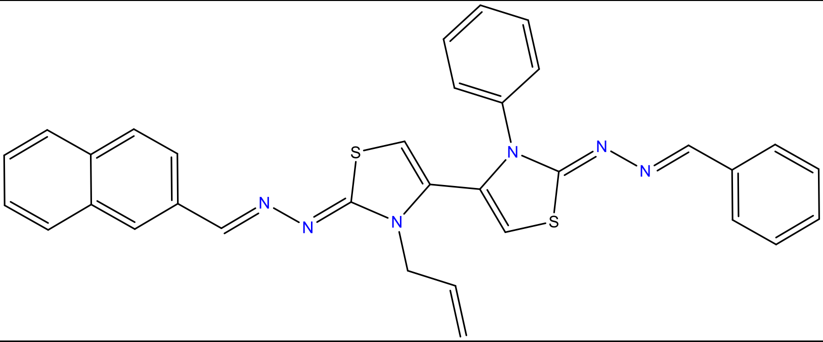	>100
814	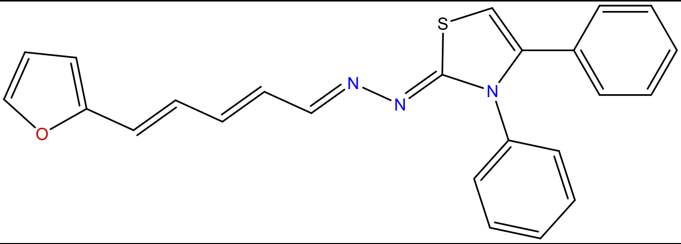	>100
306	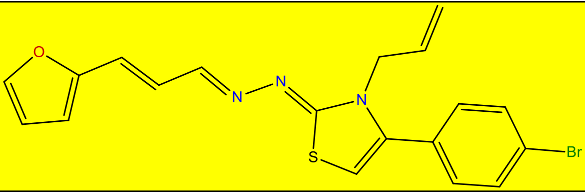	50-100
1114	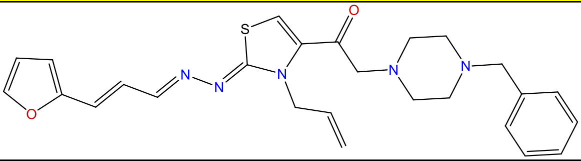	>100

901	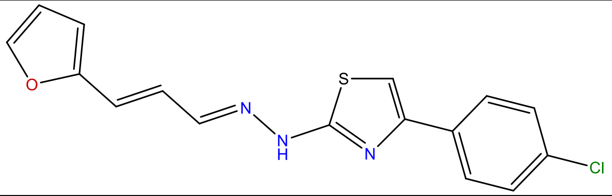	>100
913	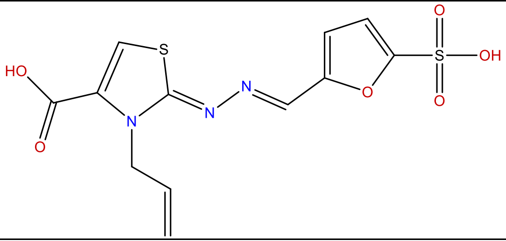	>100
813	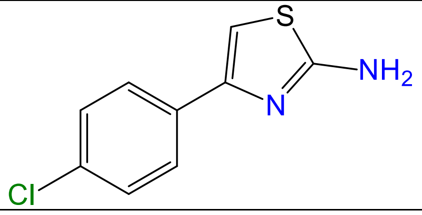	>100
784	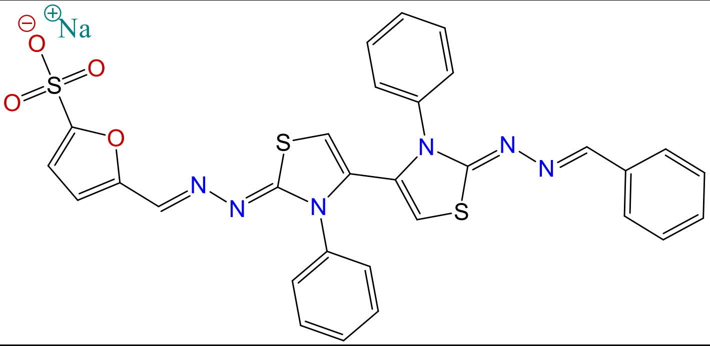	>100
782	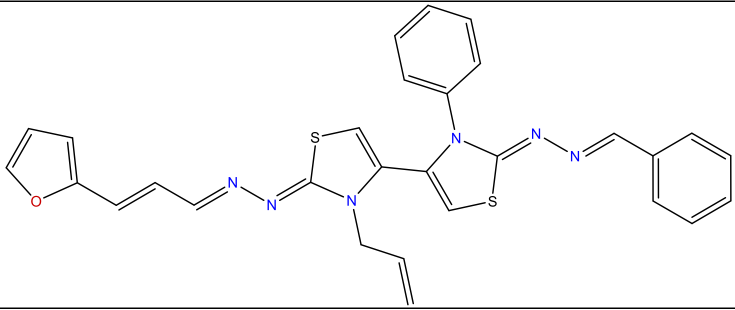	>100
1118	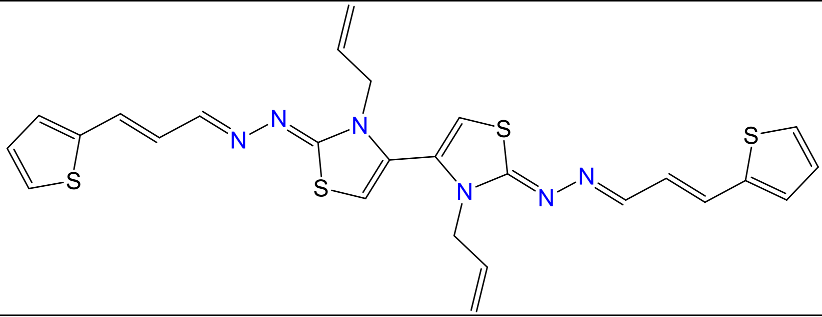	>100
1112	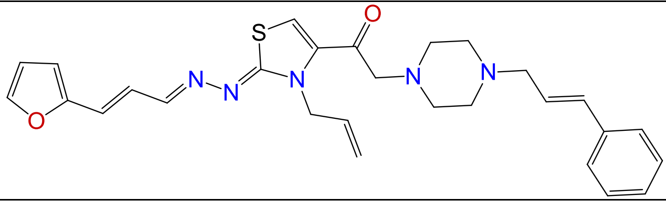	>100
789	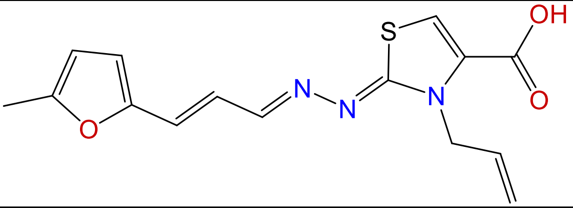	>100

910	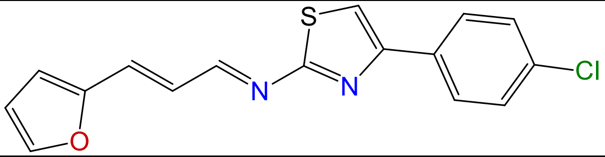	>100
1109	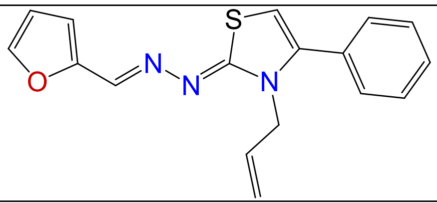	>100
1124	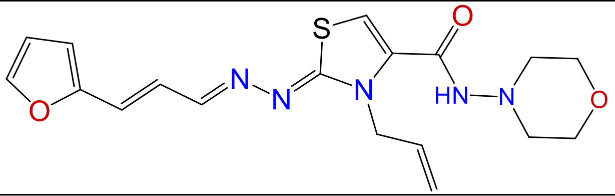	>100
1110	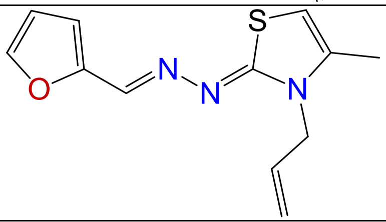	>100
1117	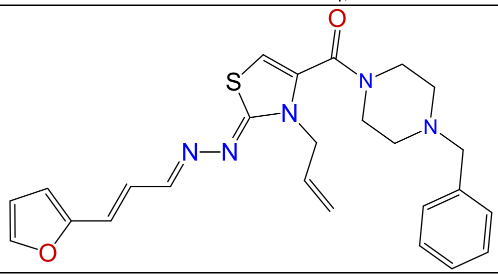	>100
1116	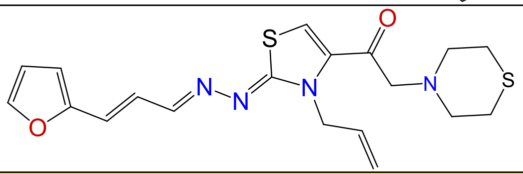	>100
144	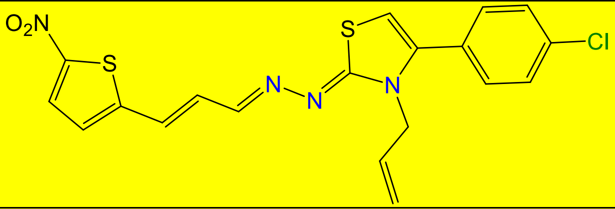	60
1134	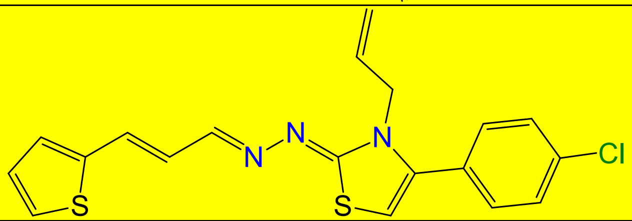	50-100
1135	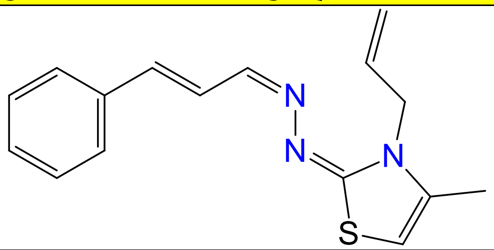	>100

1136	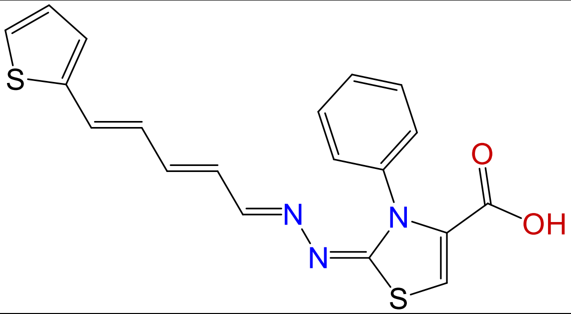	>100
1138	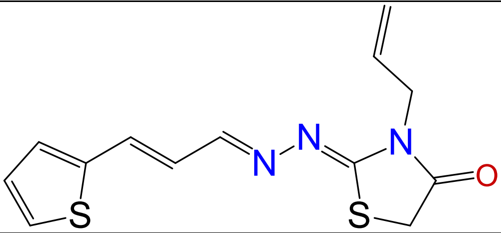	>100
312	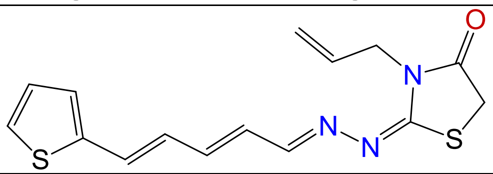	>100
304	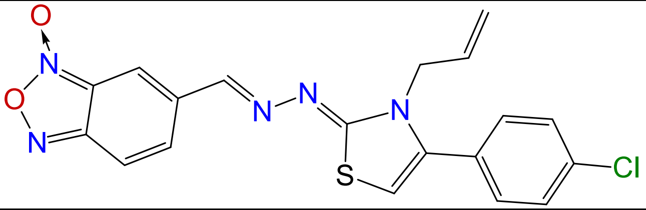	>100
282	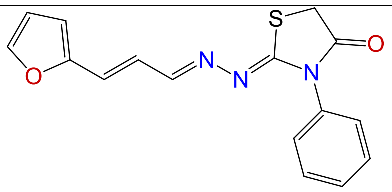	>100
131	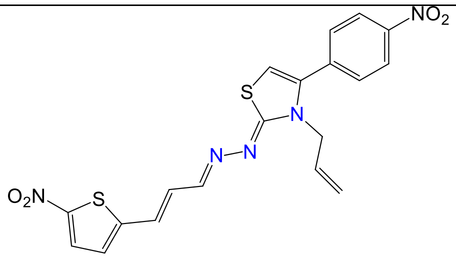	>100
908	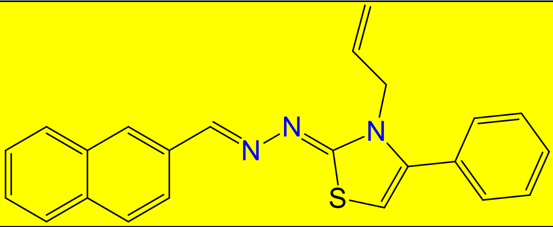	50-100
1128	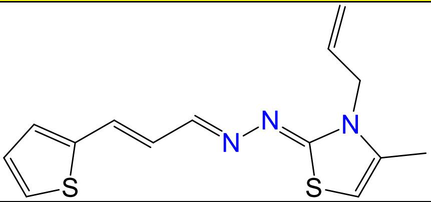	>100
1139	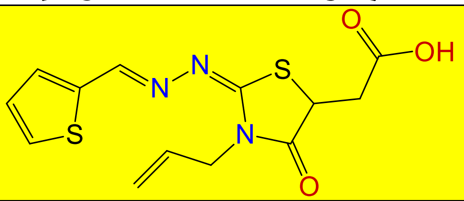	50-100

1130	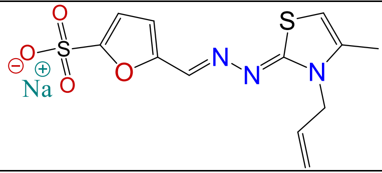	>100
313	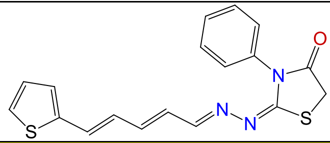	>100
875	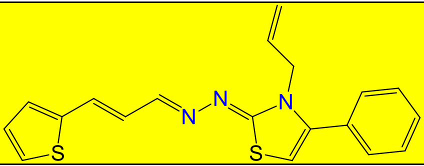	50-100
876	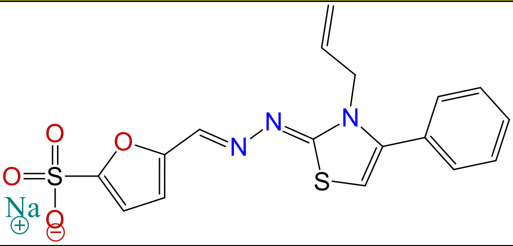	>100
1131	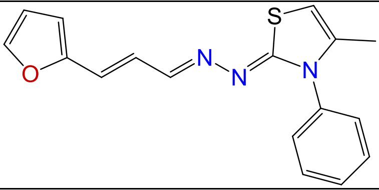	>100
775	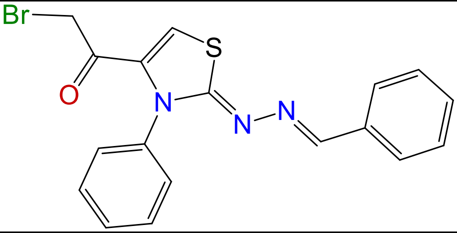	>100
1132	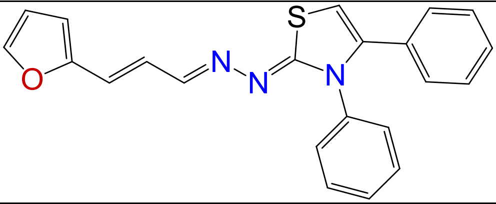	>100
1137	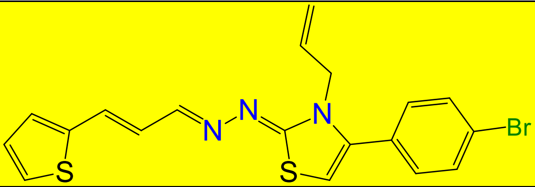	50-100
270	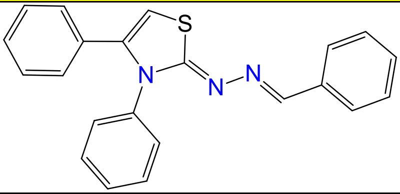	>100
276	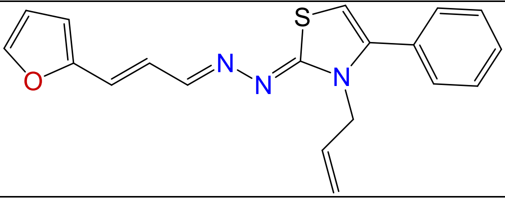	>100

871	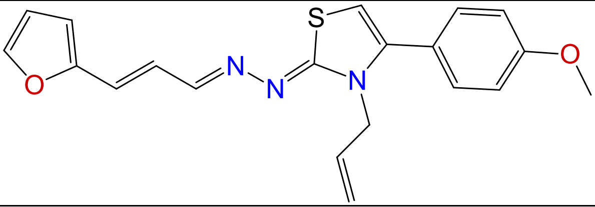	>100
904	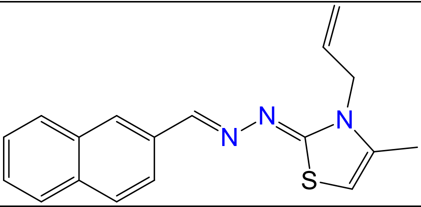	>100
311	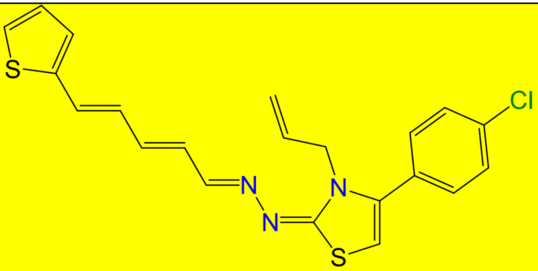	50-100
1133	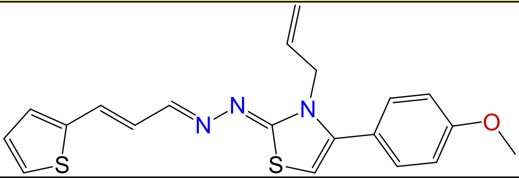	>100
907	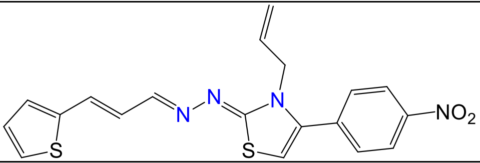	>100
293	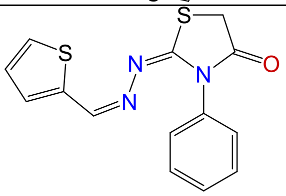	>100
1126	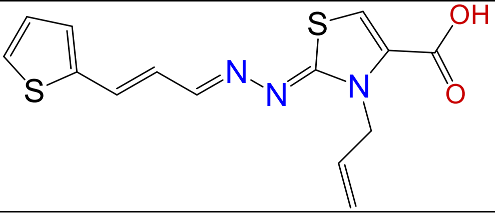	>100
	**THIADIAZOLES**	
191	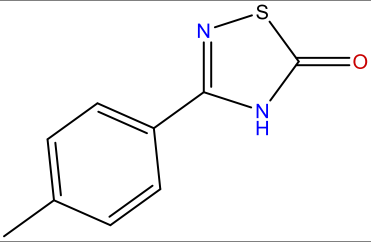	>100
292	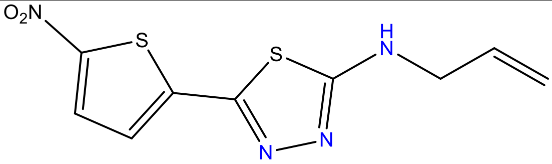	>100

287	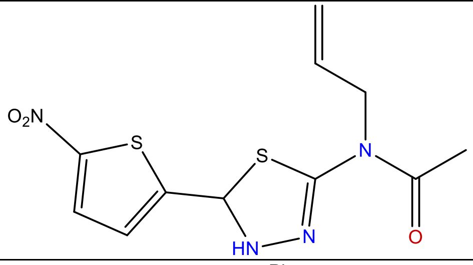 	>100
284	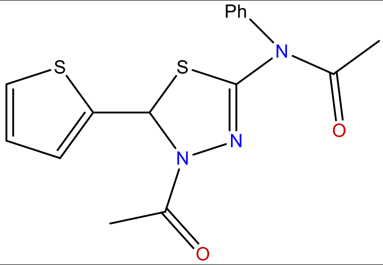 	>100
286	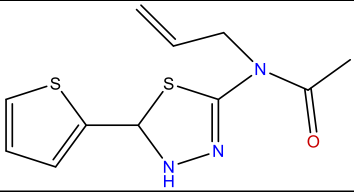 	>100
130	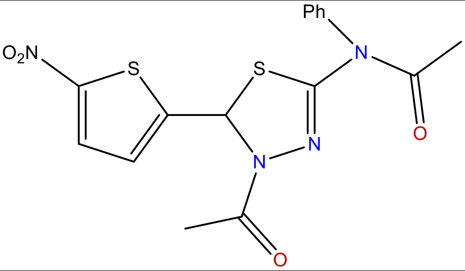 	>100
187	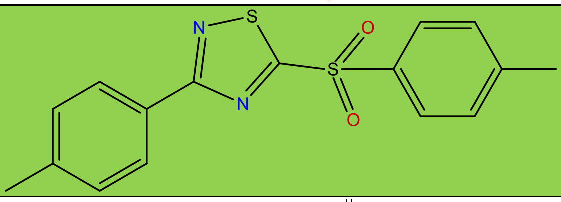 	<25
285	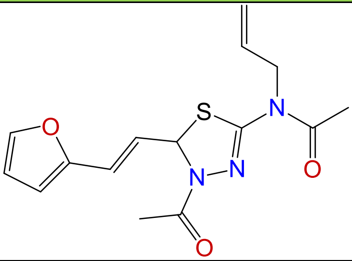 	>100
	**QUINOXALINE**	
208	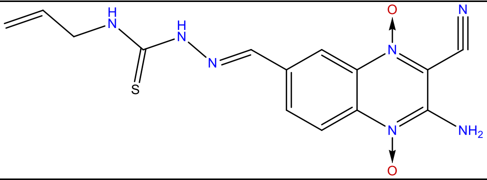 	>100
207	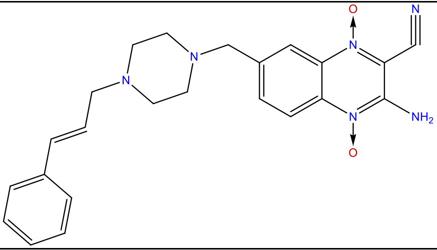 	>100
490	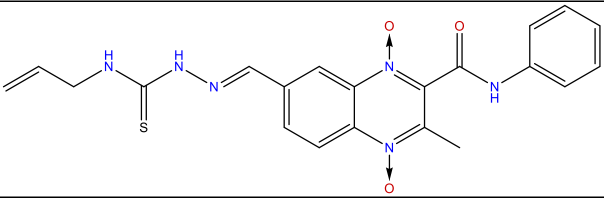 	>100

221	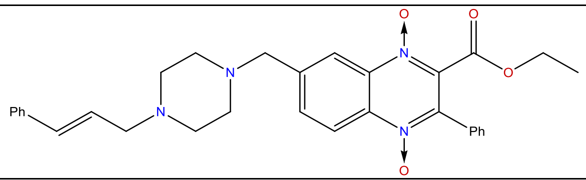	>100
655	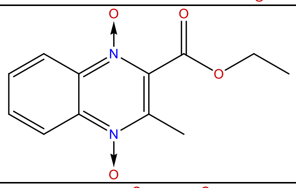	>100
658	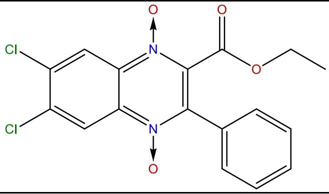	>100
327	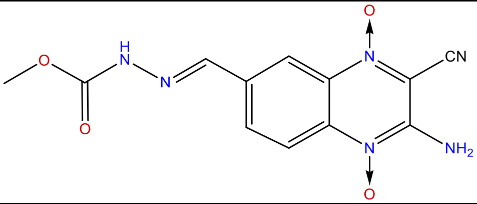	>100
656	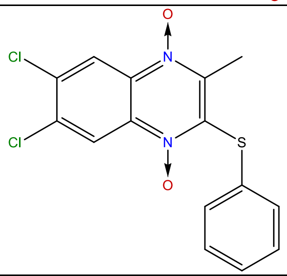	>100
297	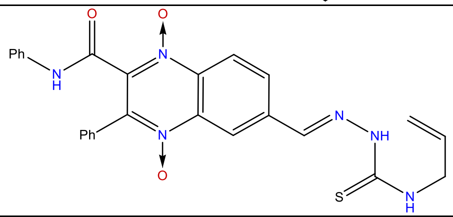	>100
657	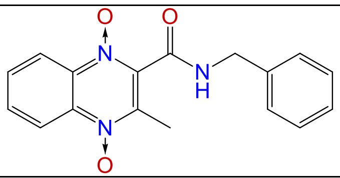	>100
652	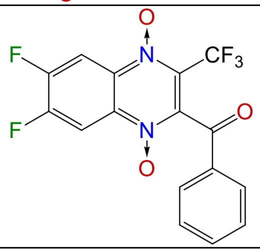	>100
649	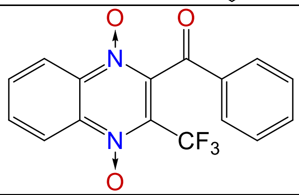	>100
303	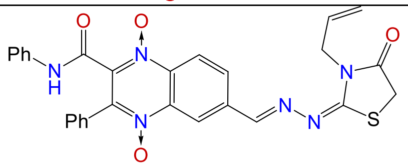	>100

301	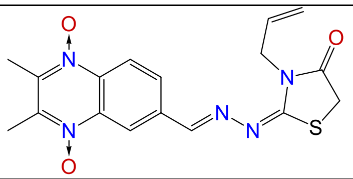	>100
302	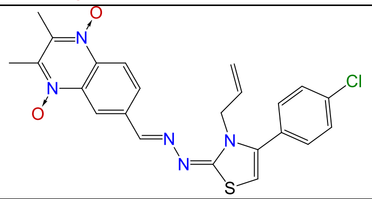	>100
362	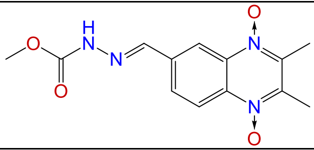	>100
220	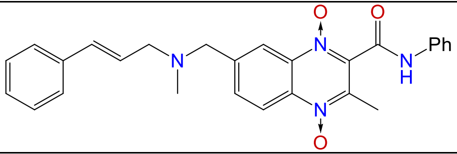	>100
1240	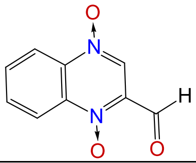	>100
492	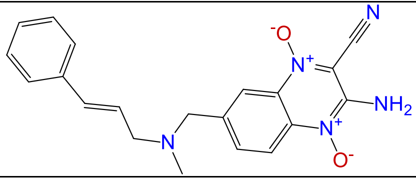	>100
489	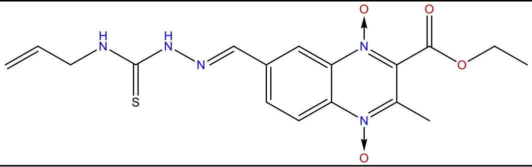	>100
364	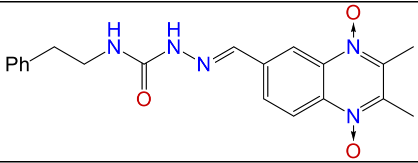	>100
	**THIOSEMCARBAZIDES**	
142	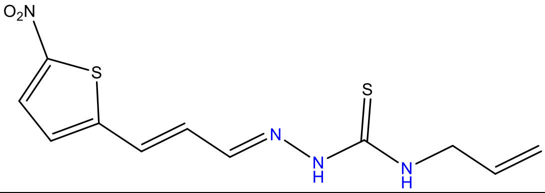	>100
277	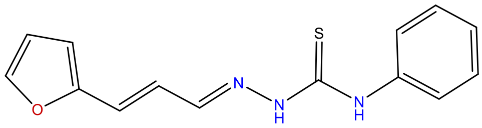	>100
278	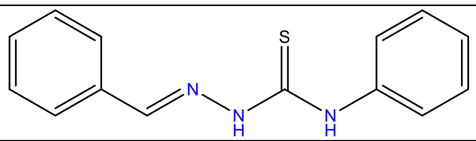	>100
283	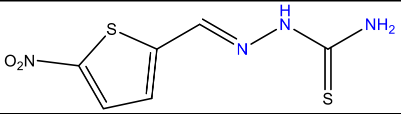	>100

135	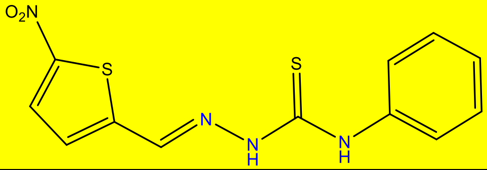	50
911	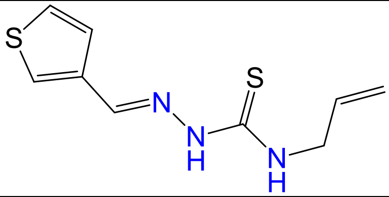	>100
900	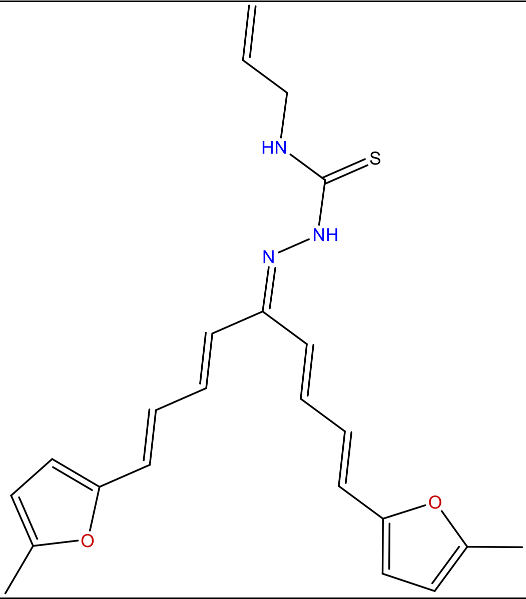	>100
136	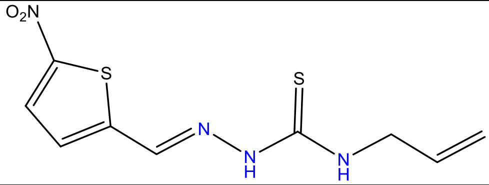	>100
263	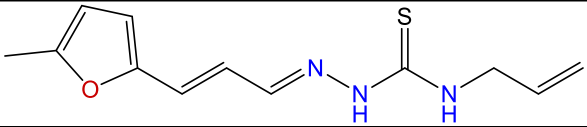	>100
281	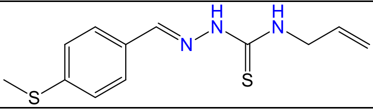	>100
788	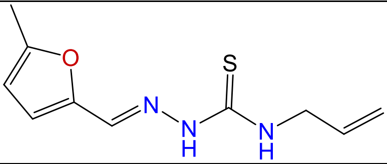	>100
307	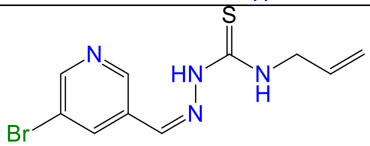	>100
308	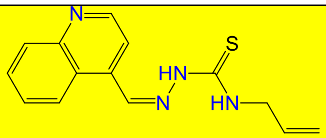	50-100
279	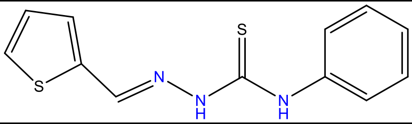	>100
261	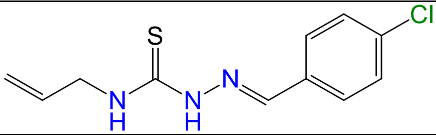	>100

280	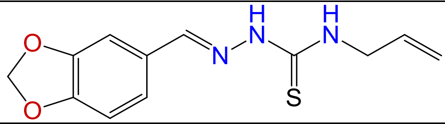	>100
291	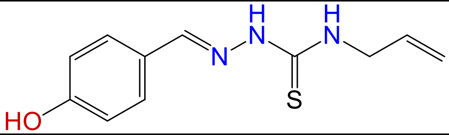	>100
787	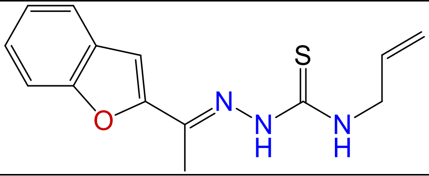	>100
296	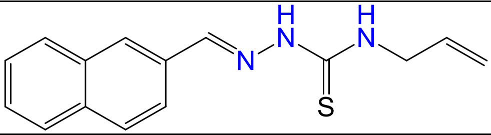	>100
310	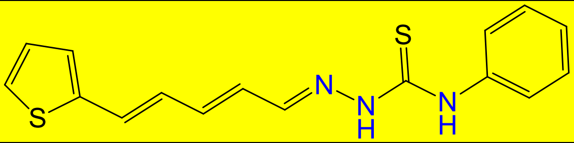	50-100
54	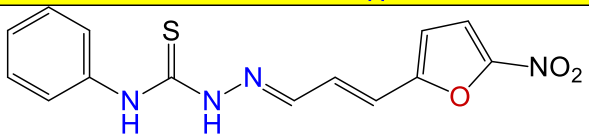	>100
69	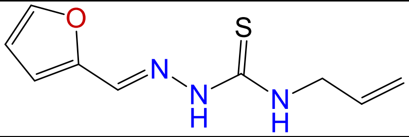	>100
300	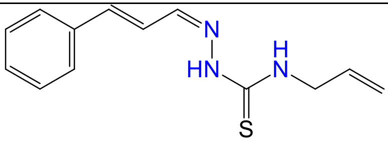	>100
	**STEROIDS**	
1272	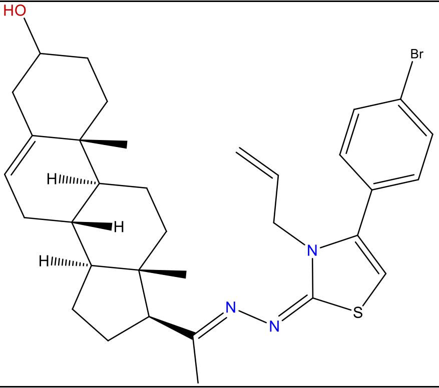	>100
1125	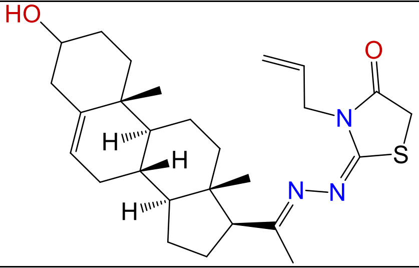	>100
1259	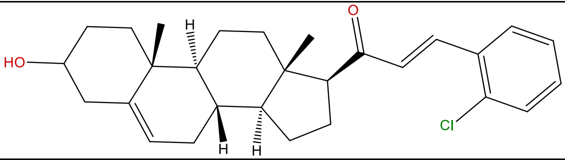	>100

1154	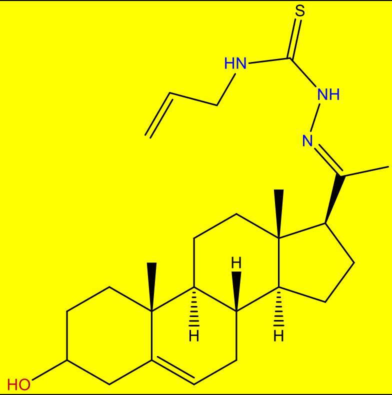	50-100
1258	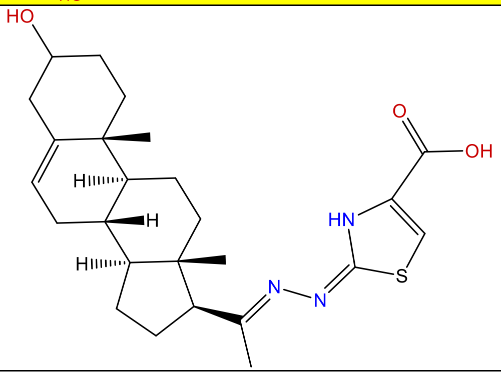	>100
1279	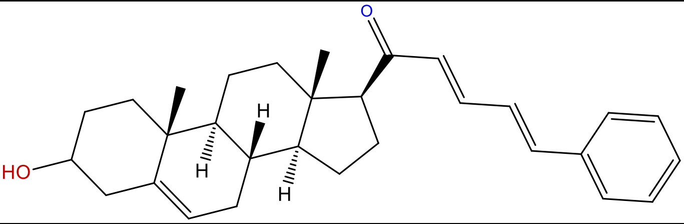	>100
**THIADIAZINES AND PRECURSORS**		
124	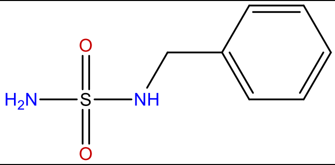	>100
120	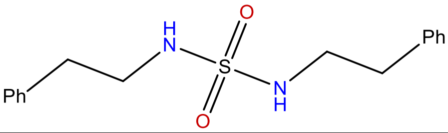	>100
127	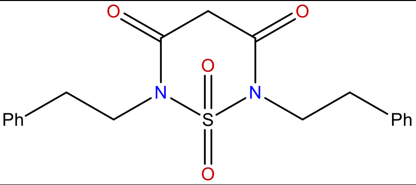	>100
723	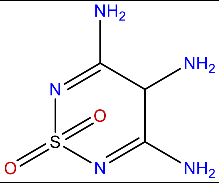	>100
128	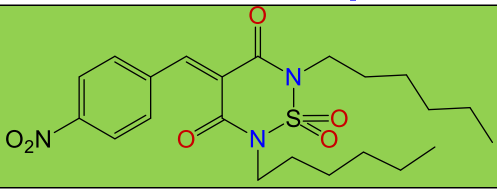	<25
123	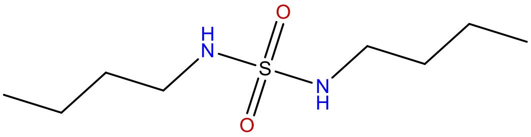	>100

126	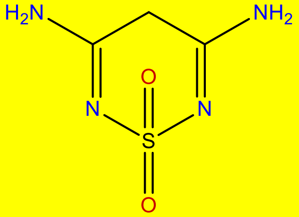	100
114	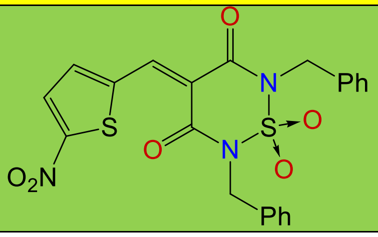	<25
706	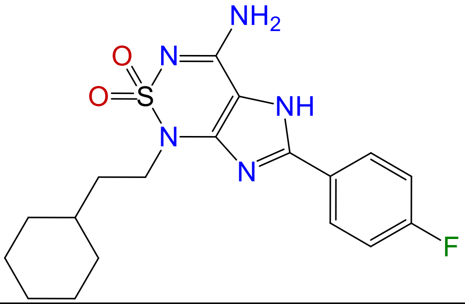	>100
707	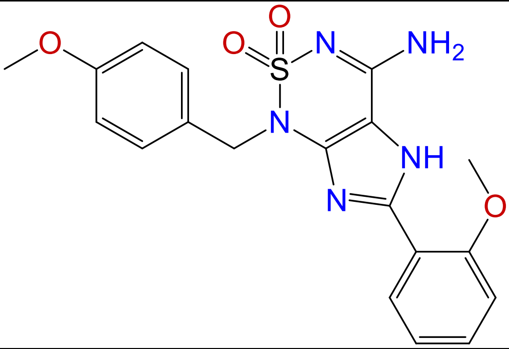	>100
1165	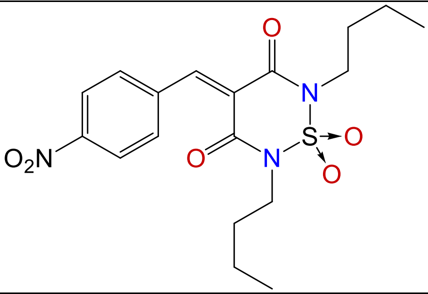	>100
1148	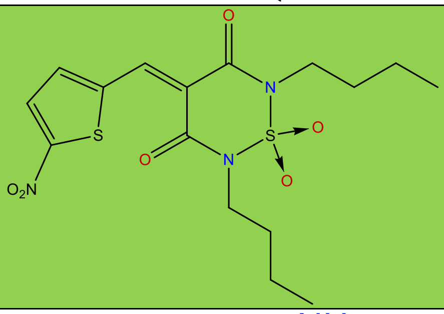	15
117	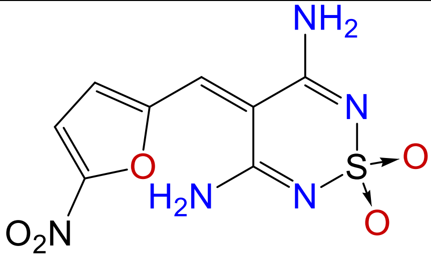	>100

358	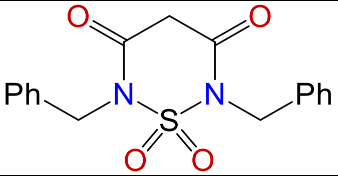	>100
116	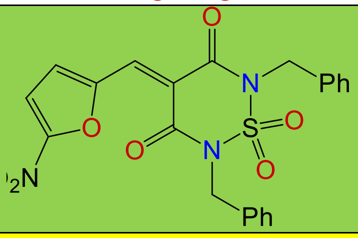	<25
121	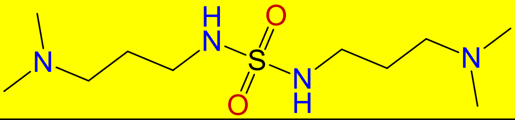	50
125	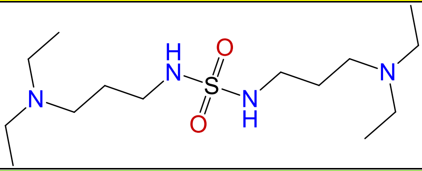	>100
110	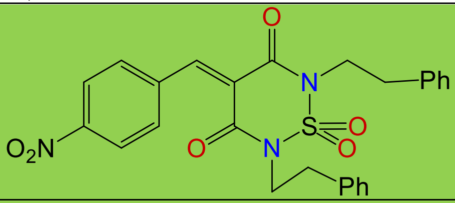	7
115	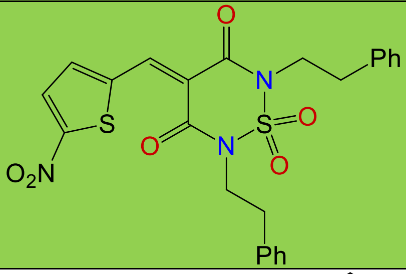	8
113	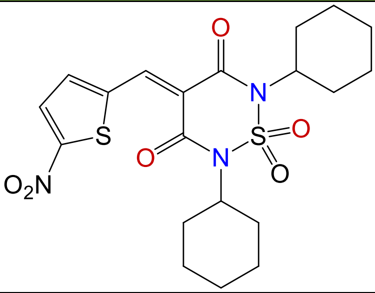	>100
118	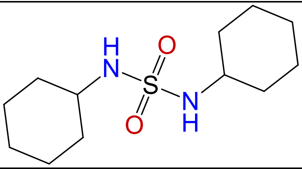	>100
119	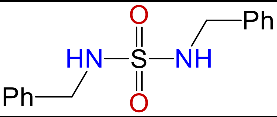	>100
111	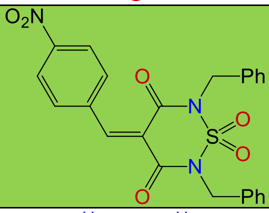	<25
716	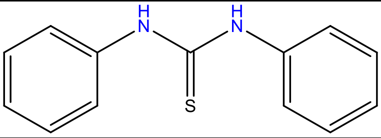	>100
	**SELENOCOMPOUNDS**	

1097	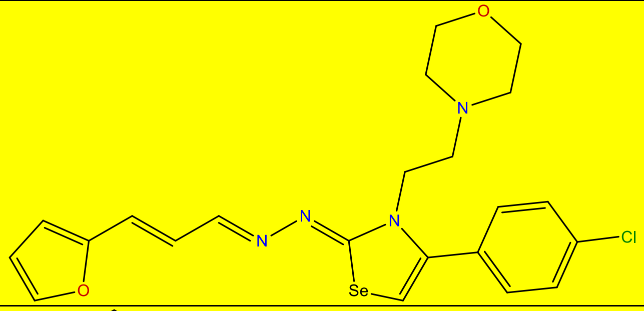	50-100
1147	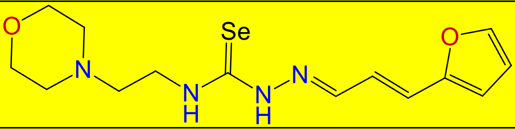	50-100
1222	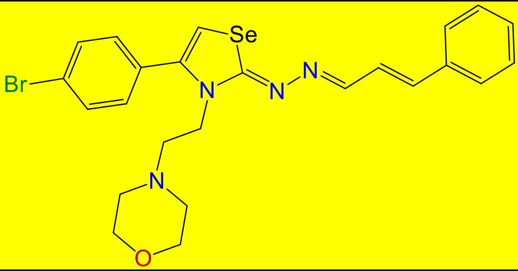	50-100
	**HYDRAZINES**	
903	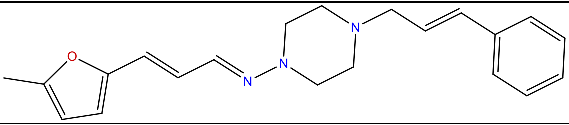	>100
717	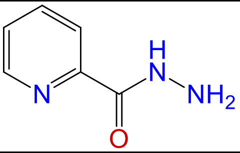	>100
874	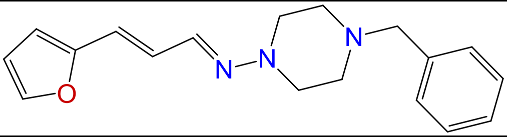	>100
1140	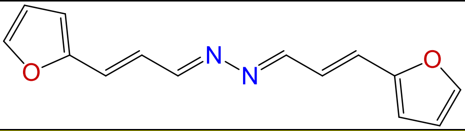	>100
878	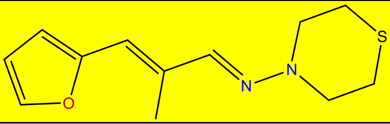	50-100
701	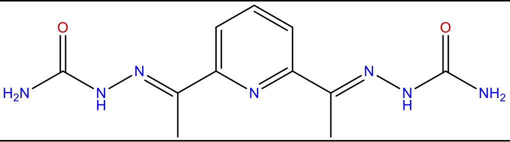	>100
675	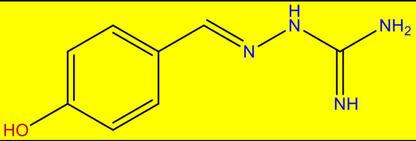	50-100
912	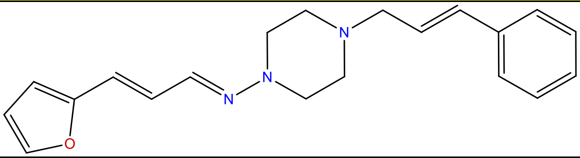	>100

1102	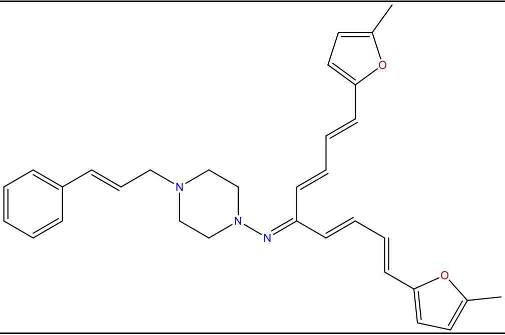	>100
1249	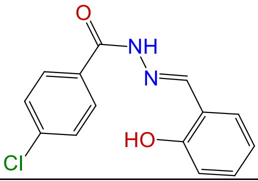	>100
1250	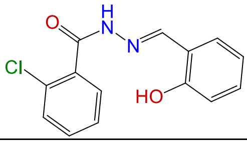	>100
777	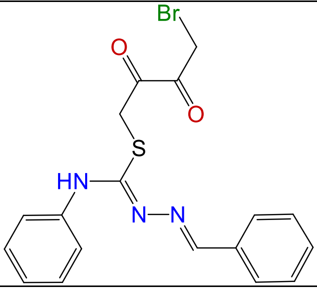	>100
397	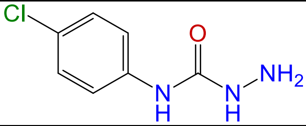	>100
616	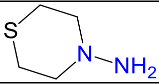	>100
1105	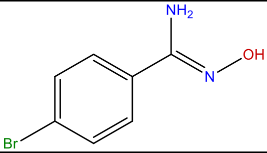	>100
1107	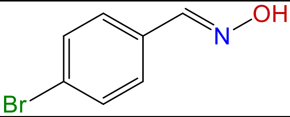	>100
1106	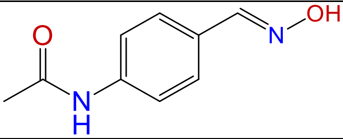	>100
1113	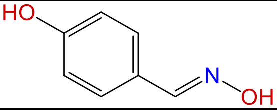	>100
711	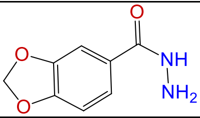	>100
1120	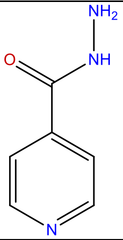	>100
	**CURCUMIONOIDS**	

906	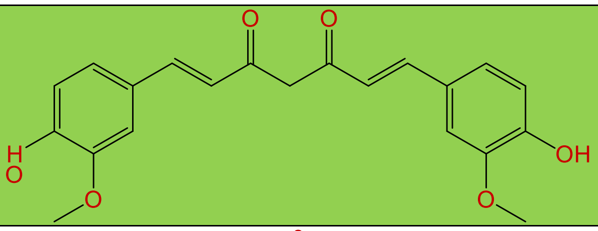	35
807	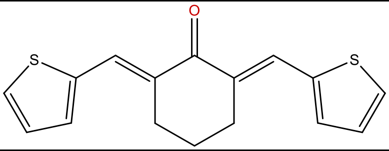	>100
1246	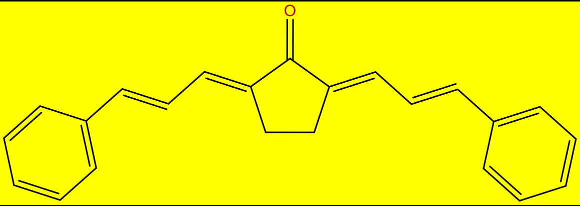	50-100
1019	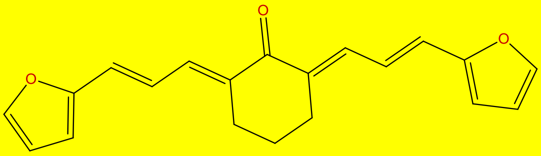	50-100
808	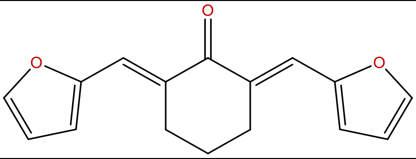	>100
798	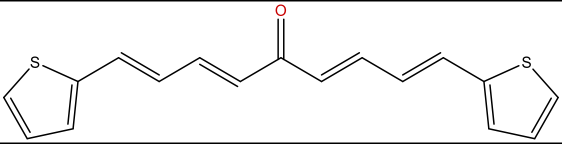	>100
797	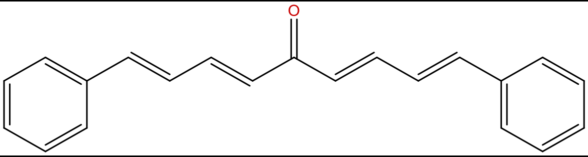	>100
1245	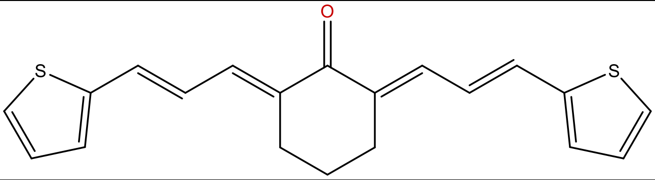	>100
1247	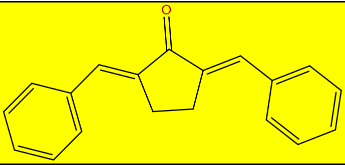	50-100
1223	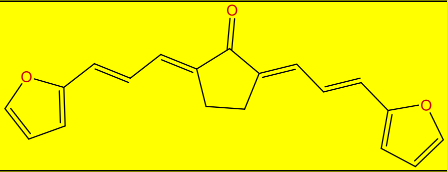	50-100
1284	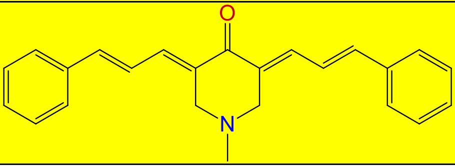	50-100
357	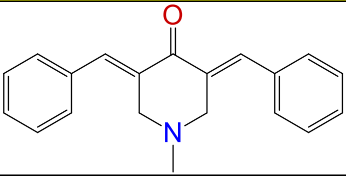	>100

1285	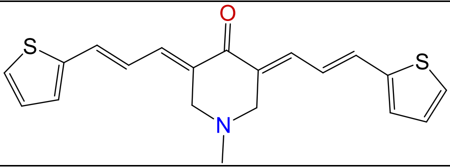	>100
1286	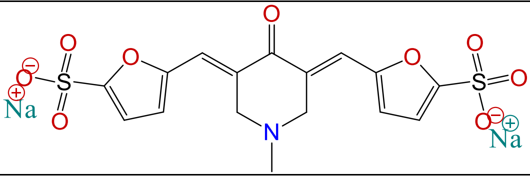	>100
1269	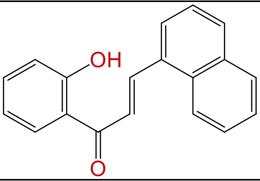	>100
1266	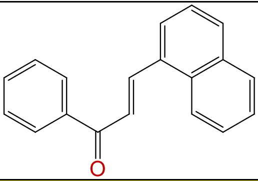	>100
881	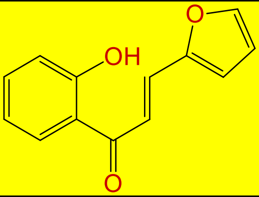	100
1254	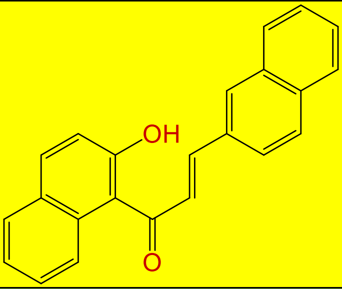	50
1267	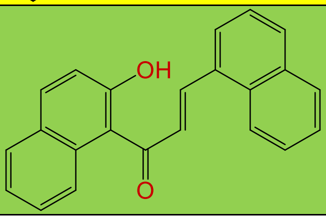	35
1155	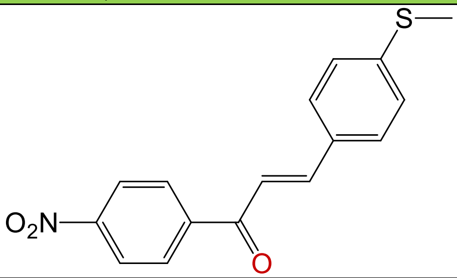	>100
1248	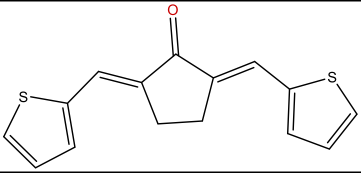	>100
796	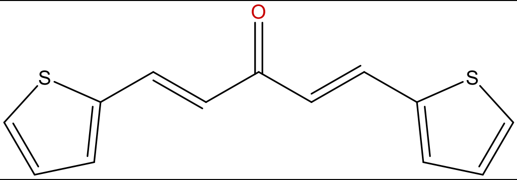	>100

1018	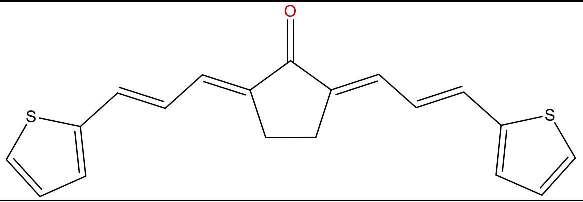	>100
793	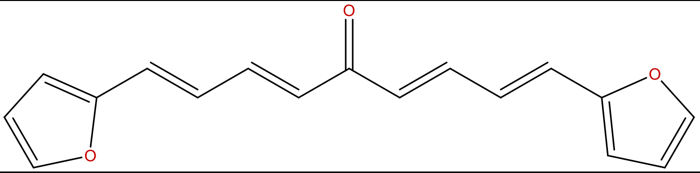	>100
809	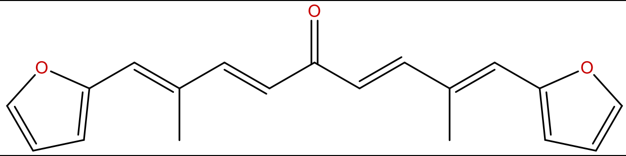	>100
1083	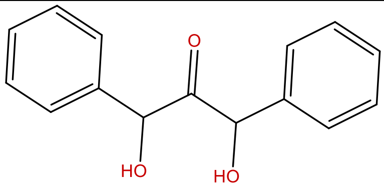	>100
1108	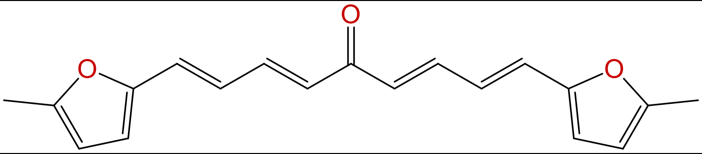	>100
1281	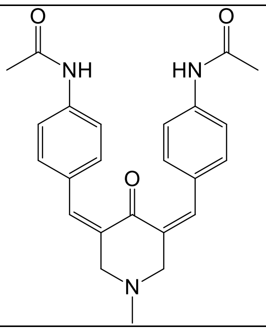	>100
1282	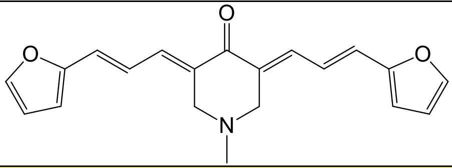	>100
799	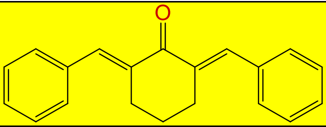	100
915	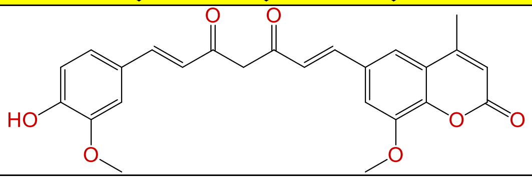	>100
148	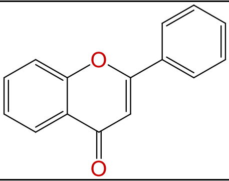	>100
800	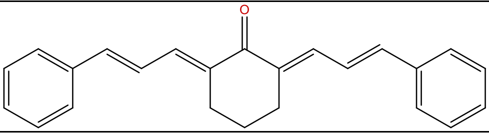	>100
	**INDAZOLES**	

505	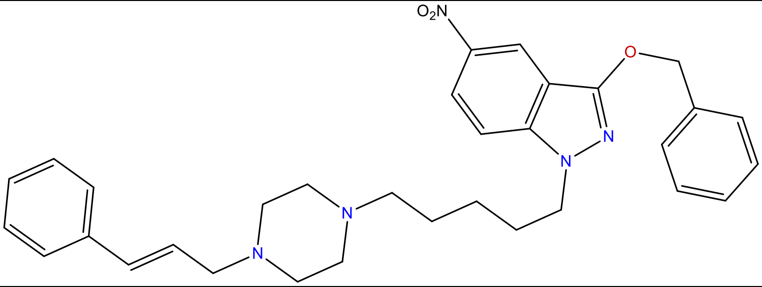	>100
500	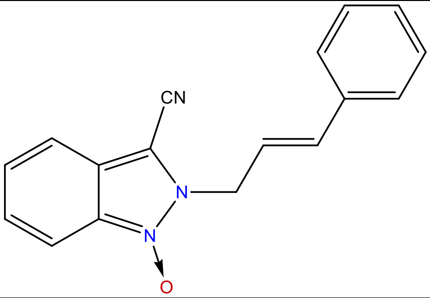	>100
469	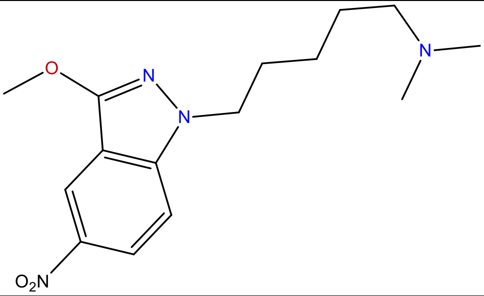	>100
518	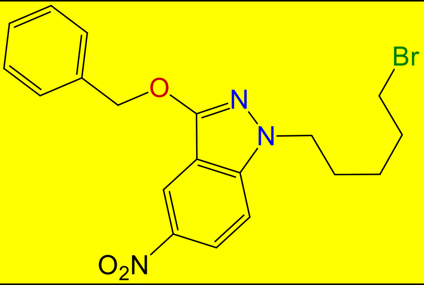	100
517	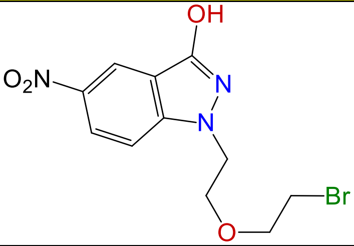	>100
456	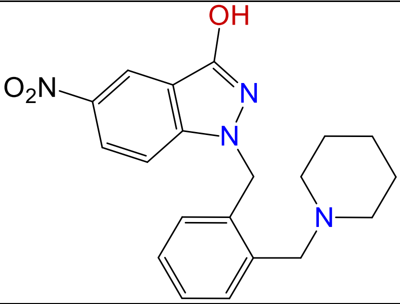	>100
516	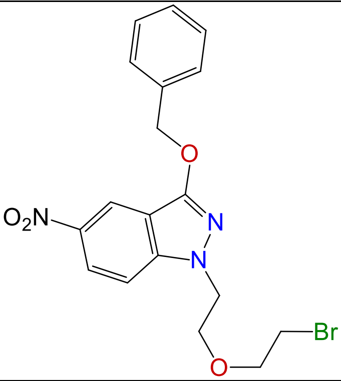	>100

501	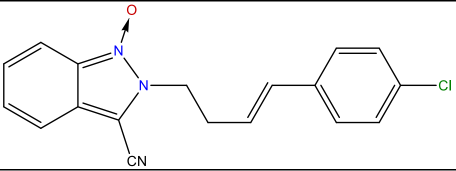	>100
504	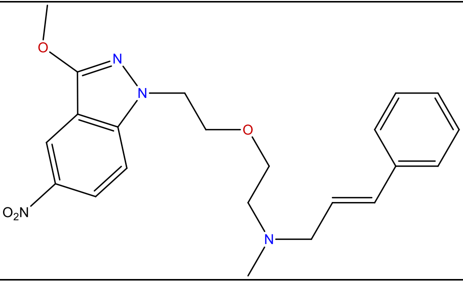	>100
508	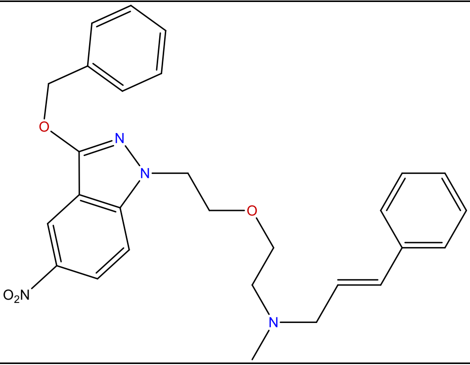	>100
480	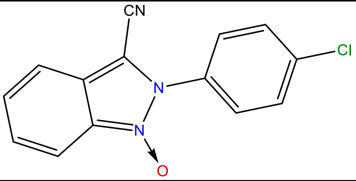	>100
507	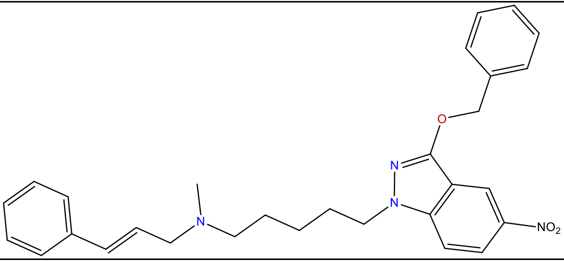	>100
514	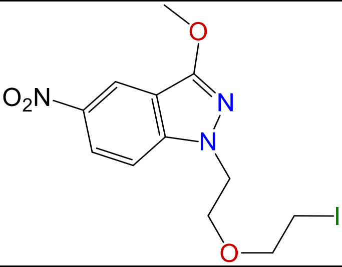	>100
642	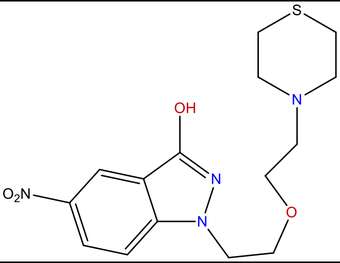	>100
1121	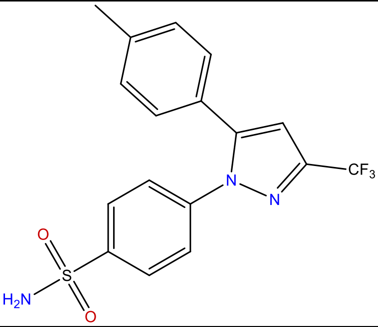	>100

474	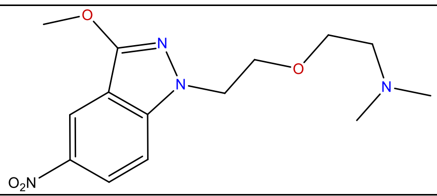	>100
202	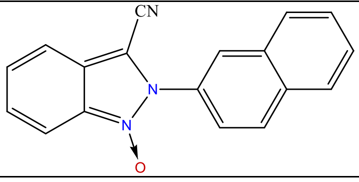	>100
198	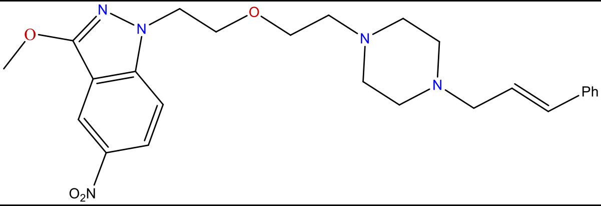	>100
459	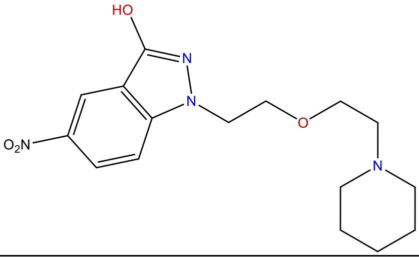	>100
483	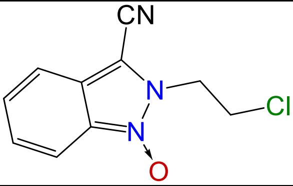	>100
199	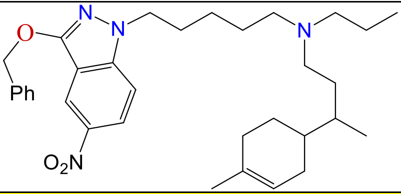	>100
481	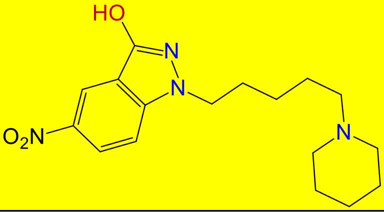	50-100
482	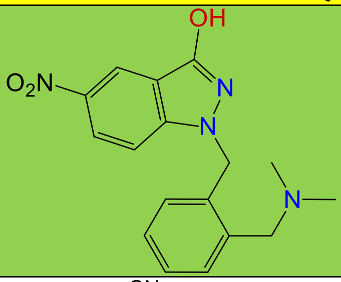	30
476	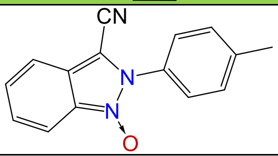	>100
643	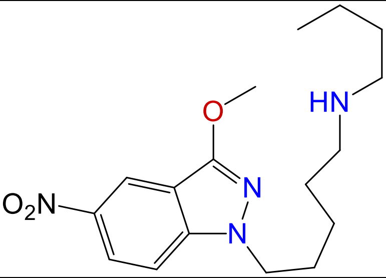	>100

637	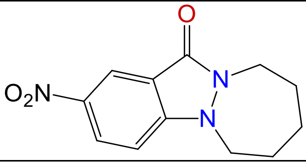	>100
477	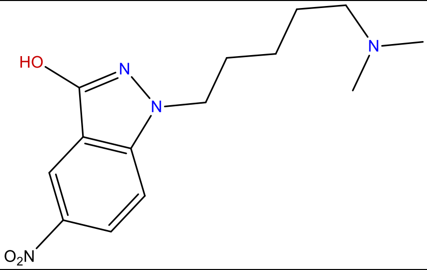	>100
	**IMIDAZOLE**	
499	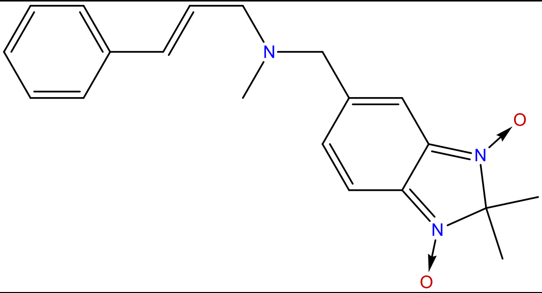	>100
633	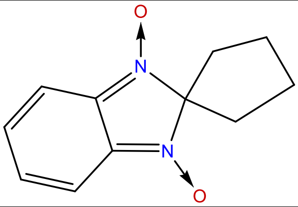	>100
545	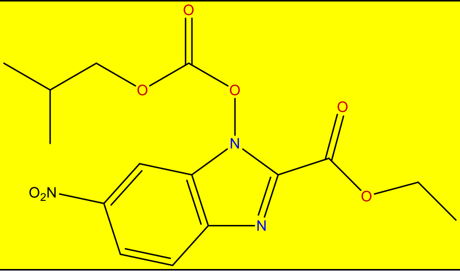	100
546	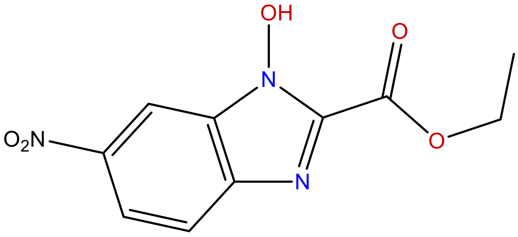	>100
660	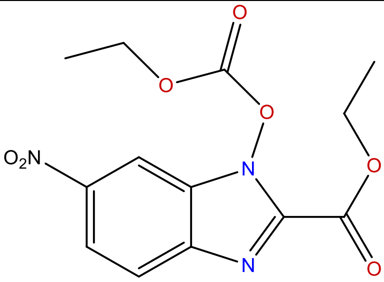	>100
550	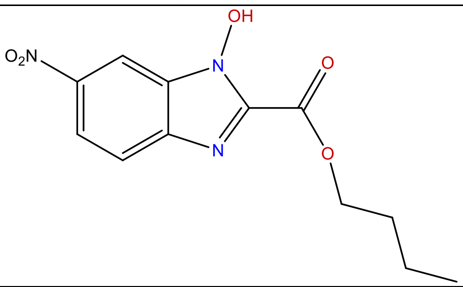	>100

553	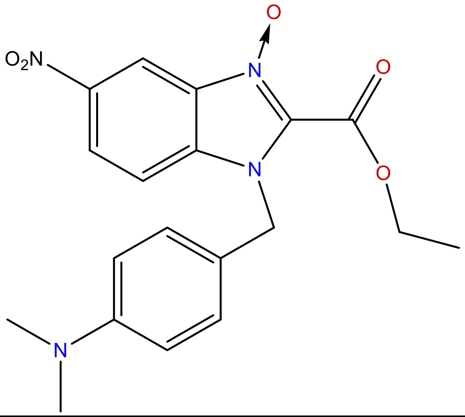	>100
712	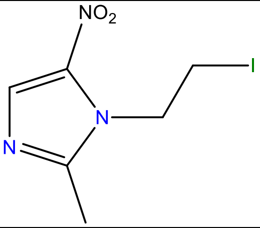	>100
109	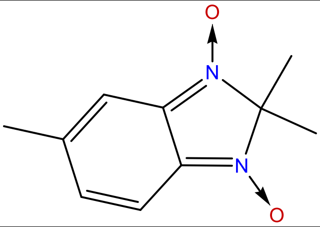	>100
1278	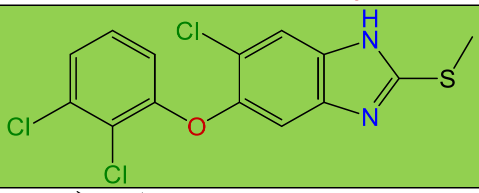	7
696	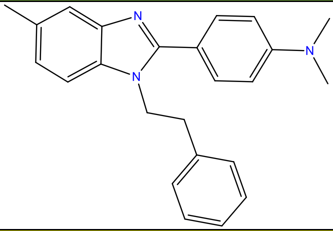	>100
532	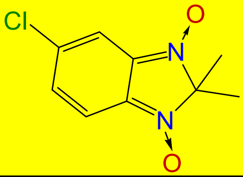	50-100
105	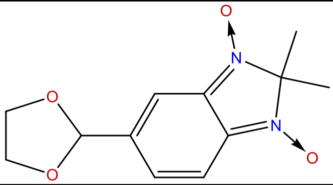	>100
	**BENZO-FUROXANES**	
498	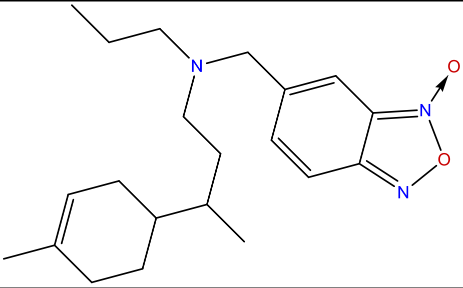	>100

704	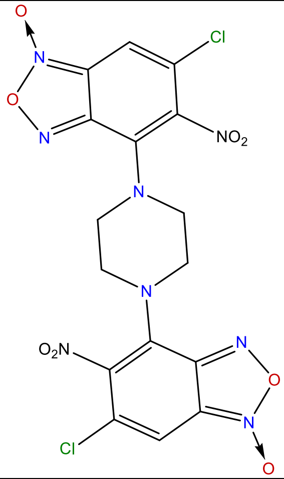	>100
609	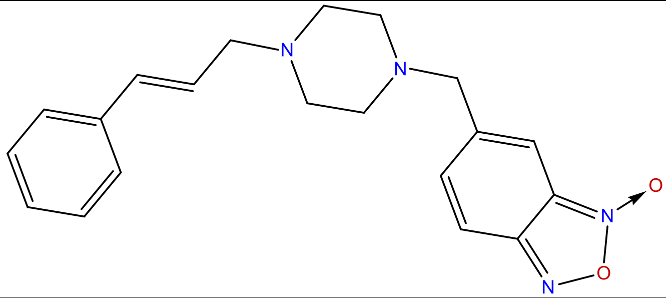	>100
175	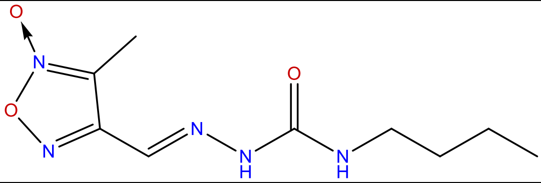	>100
627	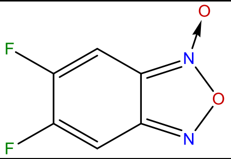	>100
38	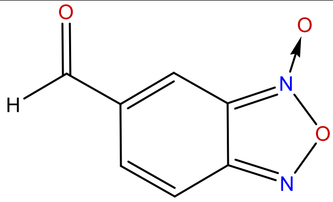	>100
671	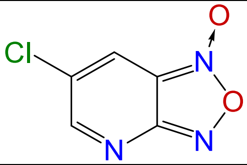	>100
31	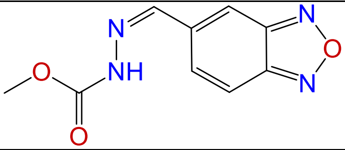	>100
12	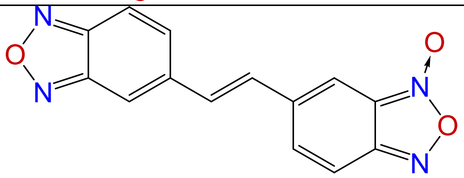	>100
174	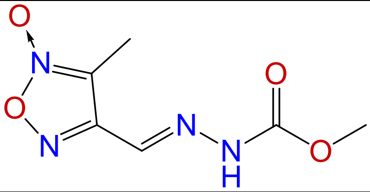	>100

169	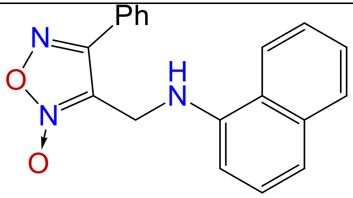	>100
650	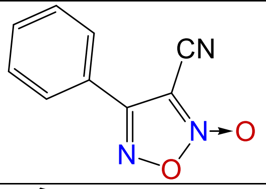	>100
584	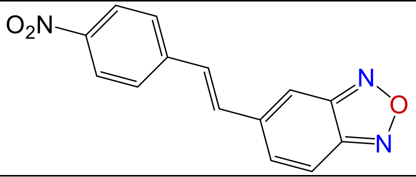	>100
1197	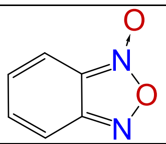	>100
173	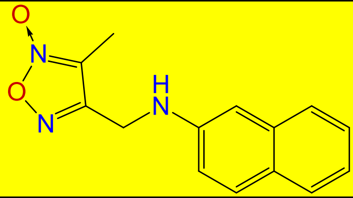	50-100
159	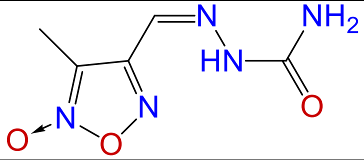	>100
1283	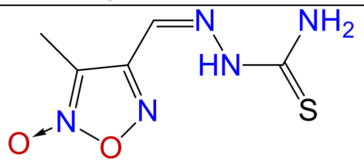	>100
352	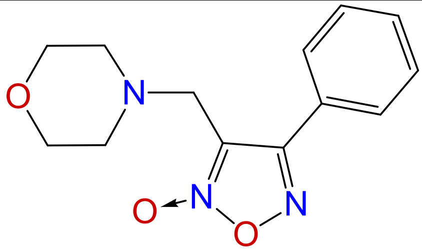	>100
172	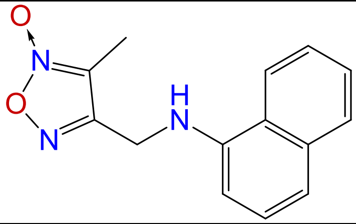	>100
824	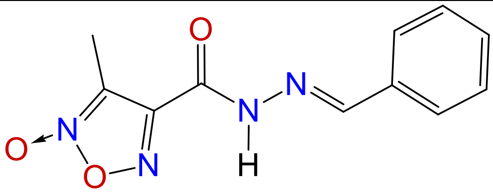	>100
41	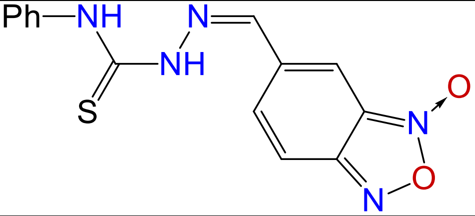	>100

TRIAZINE		
219	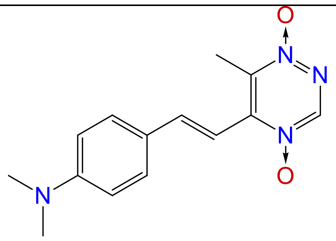	>100
211	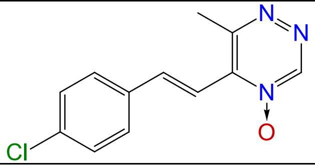	>100
360	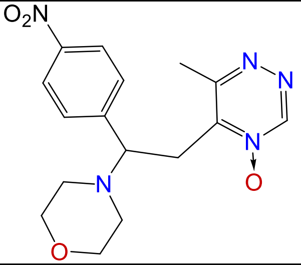	>100
365	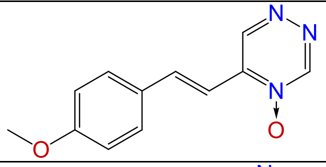	>100
359	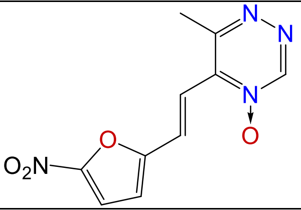	>100
213	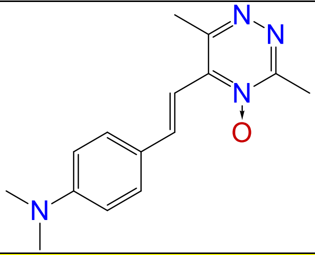	>100
361	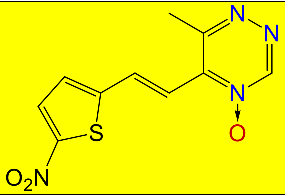	100
218	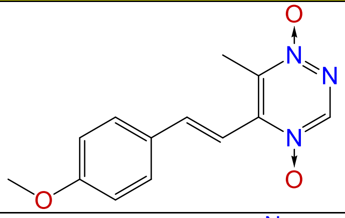	>100
216	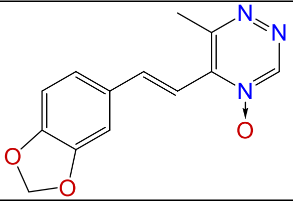	>100
214	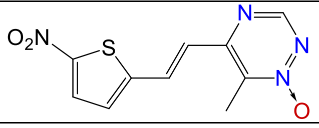	>100

215	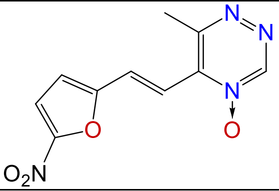	>100
641	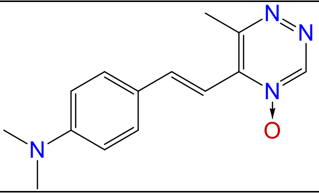	>100
**PHENAZINE**		
184	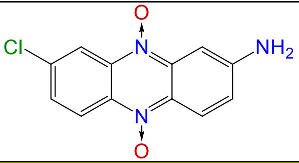	>100
1149	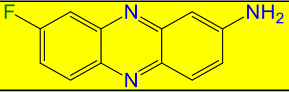	50
183	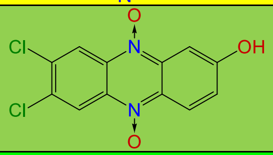	25
**NOT CLUSTERED COMPOUNDS**		
1129	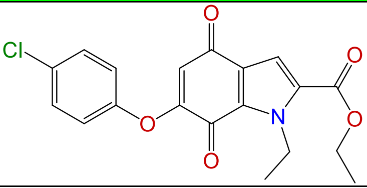	>100
1127	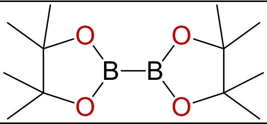	>100
497	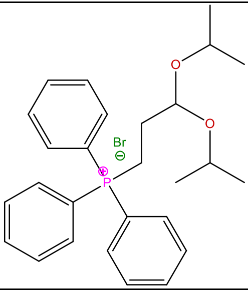	>100
237	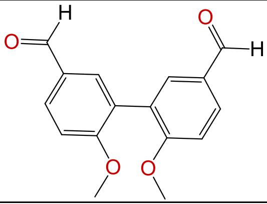	>100
579	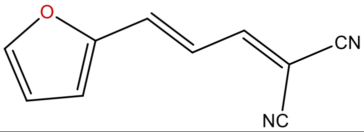	>100
227	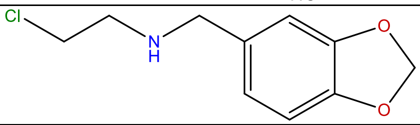	>100
1085	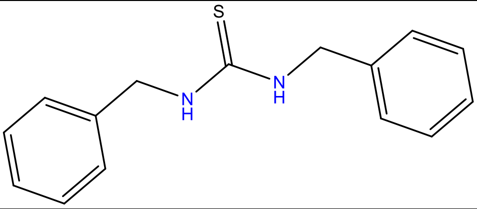	>100

1089	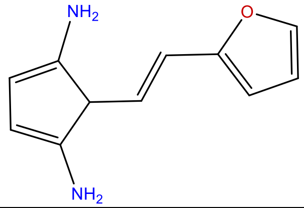	>100
581	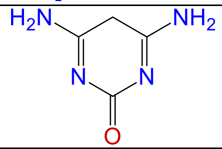	>100
1082	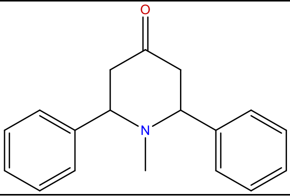	>100
1088	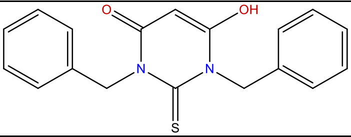	>100
239	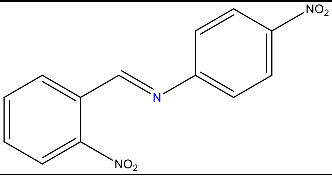	>100
431	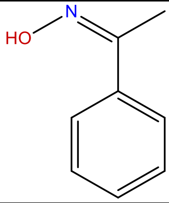	>100
1122	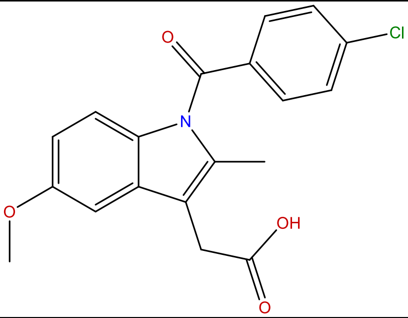	>100
242	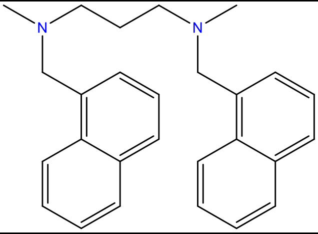	>100
1104	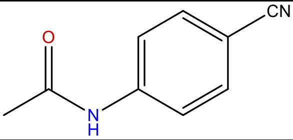	>100
245	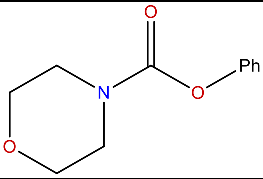	>100
1101	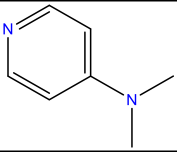	>100

379	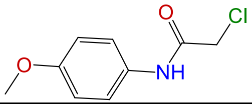	>100
659	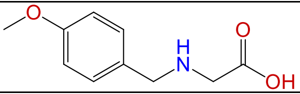	>100
50	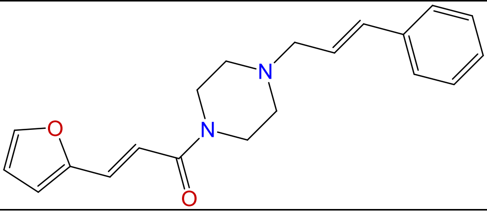	>100
1119	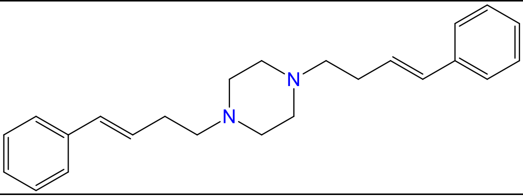	>100
51	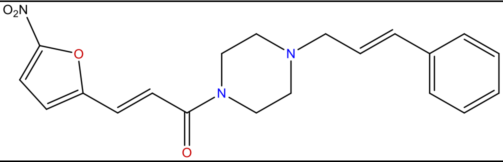	>100
60	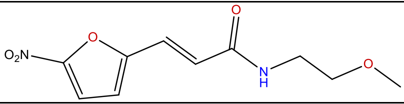	>100
879	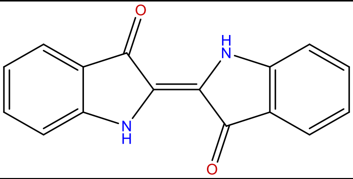	>100
573	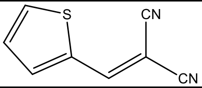	>100
575	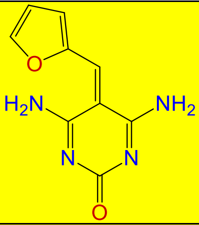	50-100
1084	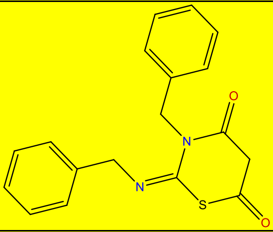	50-100
670	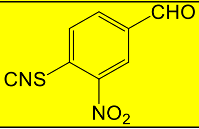	100
698	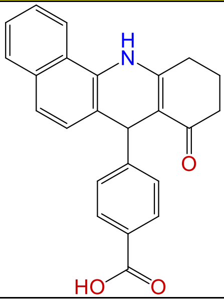	>100

699	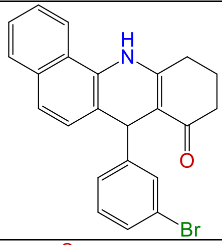	>100
617	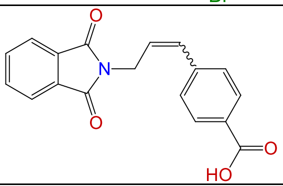	>100
66	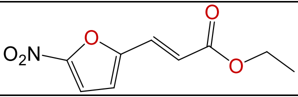	>100
811	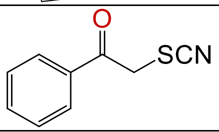	>100
1196	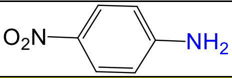	>100
608	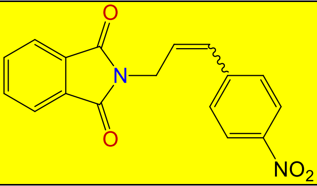	100
472	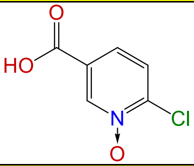	>100
769	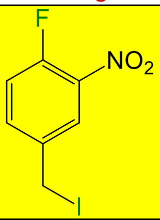	100
651	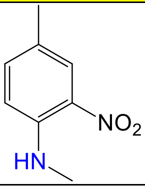	>100
1198	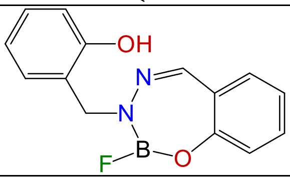	>100

Figure 1S docking estudies.

<u>Figure 1.2S</u>

A) Top (left) and side (right) view of the content of the asymmetric unit with one SO4 molecule (red and yellow) per protonomer. Dimer A-D:blue tints; dimer B-C: green tints; dimer E-F: yellow tints). Black rectangle highlights the dimer shown in B)

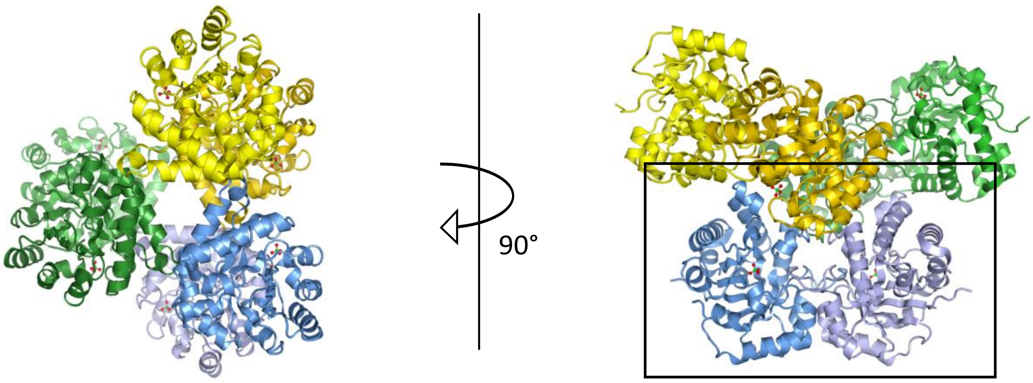

B) Close-up view of dimer A-D with the two SO4 molecules in the active site of each monomer (red and green). Red rectangle highlights the active site of FhTIM shown in C), left panel

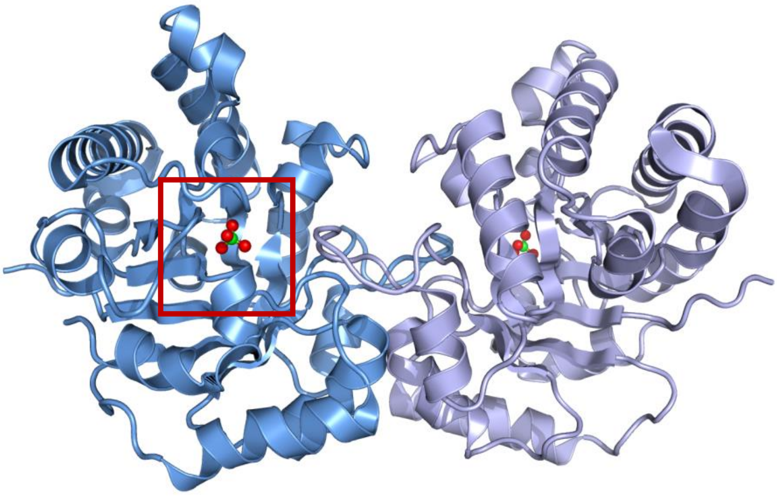

C) Close-up comparison of the active site of chain A of FhTIM (left) with the active site of chain A of *P. falciparum* TIM (PDB ID: 1LYX, right) with their ligands SO4 and phosphoglycolate (PGA), respectively. Dashed lines display hydrogen bonds of the ligand with neighbouring residues/water molecules. Red sphere corresponds to water molecule 465 of FhTIM, chain A. Coloring scheme for the ligands' atoms is: red=oxygen, yellow=phosphorus, green=sulfur, light gray=carbon, orange=chloride.

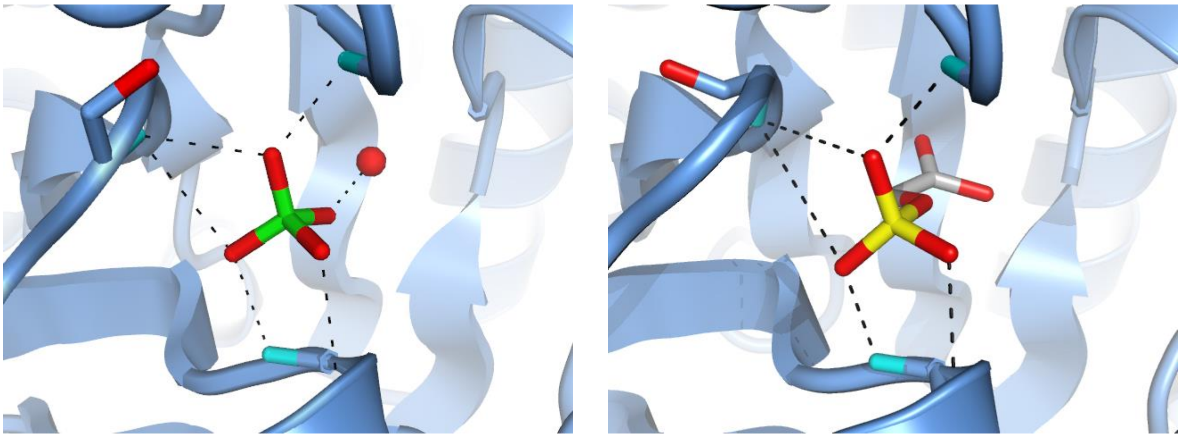

<u>Figure 1.2S</u>

Best solution after docking of compounds 110 (A and B) and 187 (C and D) on the FhTIM dimer. A and C: general view of the position of the compounds, rotated 45° along the x-axis when compared to figure 1B. B and D: close-up view of the position of the compounds with a display of the neighboring residues for which at least one atom lies within 4Å of the ligand. Panel B is rotated compared to panel A for clarity. Ligand coloring scheme is similar to fig 1C.

A

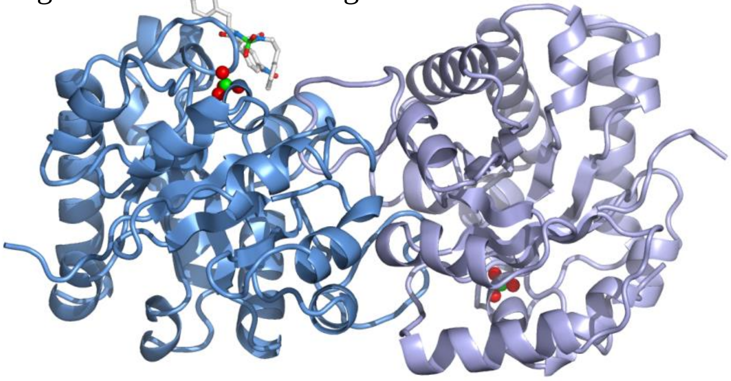

B

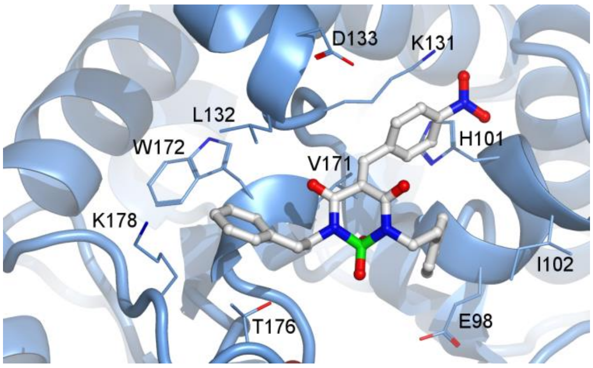

C

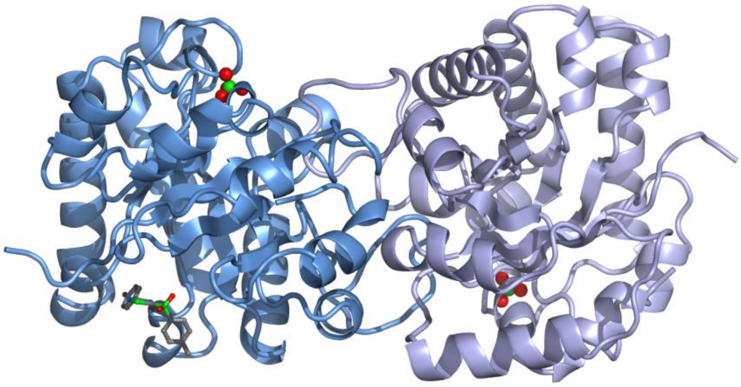

D

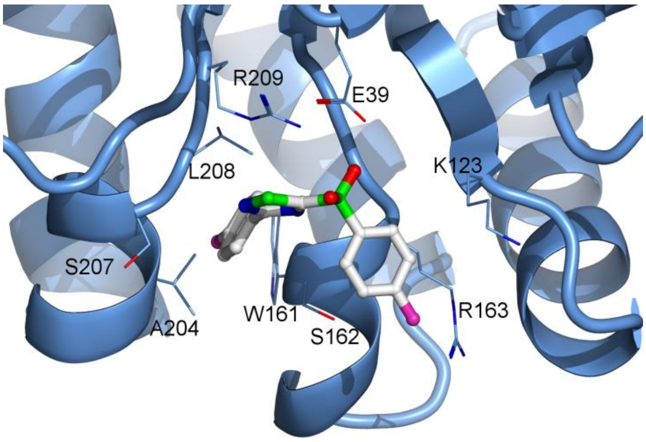

Figure 1.3S: Display of the best solutions for each compound after docking with compounds 110, 187 and 1278 on the monomeric form of FhTIM. The position of the second monomer in the dimer is displayed as a ghost to display the location of the docked compounds compared to the dimeric interface.

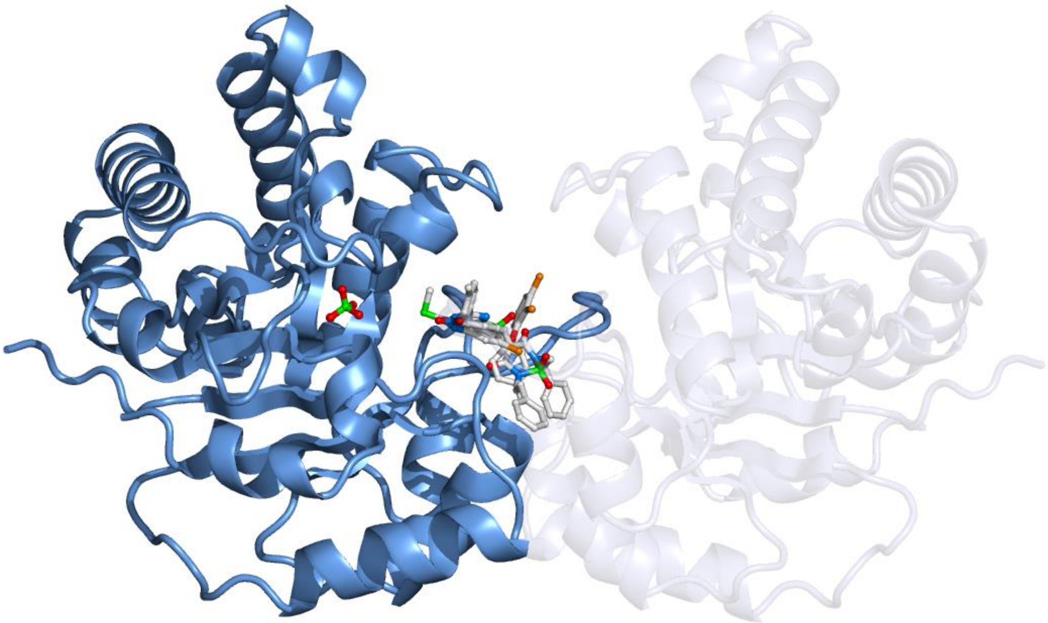

Figure 1.4S:
Best solution after docking of compounds 110 (B and C), 187 (D and E) and 1278 (F and G) on the FhTIM monomer. B, D and F: general view of the position of the compounds. C, E and G: close-up views of the position of the compounds with a display of the neighboring residues for which at least one atom lies within 4Å of the ligand. These views are rotated 90° around the y-axis compared to panels B, D and F for the sake of clarity. Ligand coloring scheme is similar to fig 1D.

B

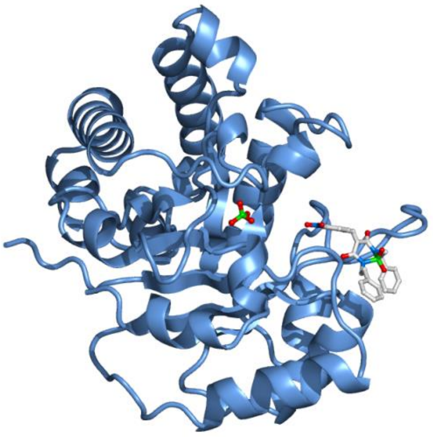

C

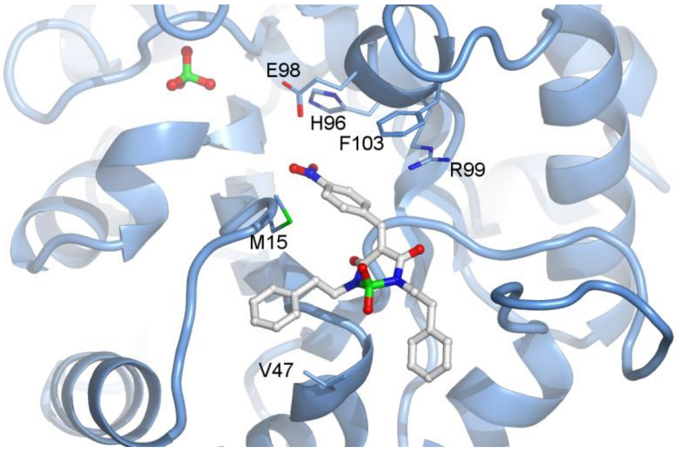

D

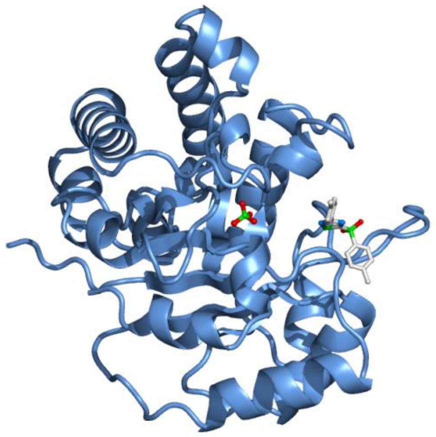

E

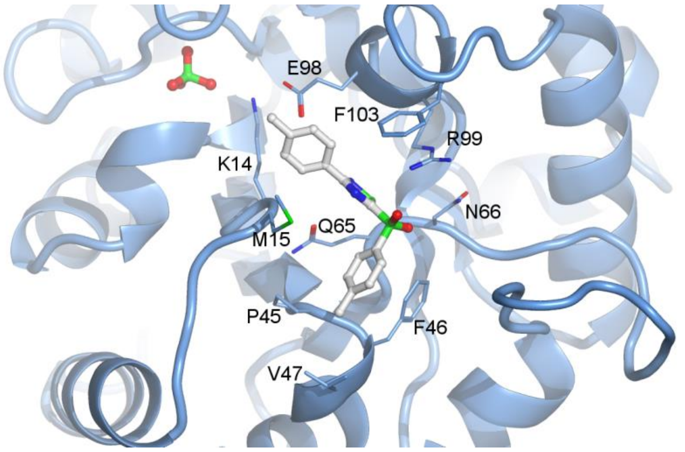
E98
F103
R99
K14
N66
Q65
M15
P45
F46
V47

F

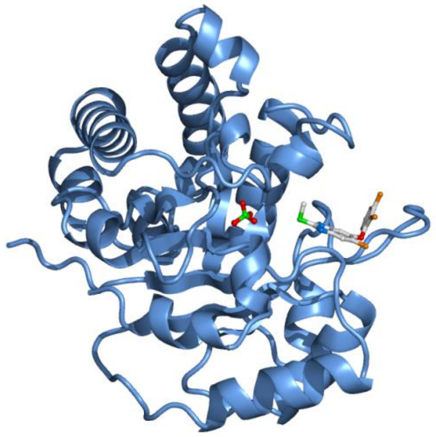

G

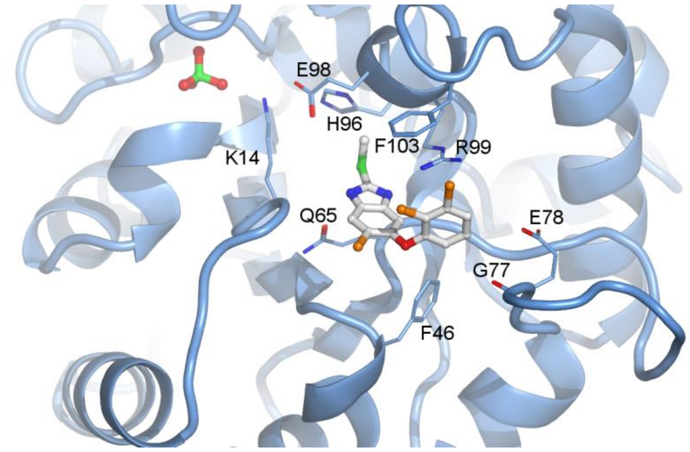
E98
H96
F103
R99
K14
Q65
E78
G77
F46

Table 2S. Severity scores used:

Somule Descriptior Key	Severity score
D = dead	4
O = overactive	1
I = Immobile	1
S = Slow	1
Deg= degenerating	4
Dark = dark	1
R = shape altered from normal (either larger or smaller)	1
Plus and minus signs indicate degrees more or less from the descriptor applied	1

Adult Descriptior Key
Dark = dark
S = Slow
O = overactive
Uncoord= uncoordinated movements
S-uncoord = uncoordinated slow movements
O-uncoord = uncoordinated fast movements
On sides = male worms are not adhering to dish with ventral sucker
Shrunk = worms are not flexing and are smaller than usual
Teg damage = outer surface (tegument of the worm) is damaged

Supporting videos for the experiments in adult of S mansoni with **187** and with nej of F. hepatica. In the videos you can see the effect in the morphology and in the parasite movement compared to the control without treatment.

			SOMULES 080116 (10 uM in replicates)						ADULTS 080316 (5 uM)						
			DESCRIPTORS			SEVERITY SC+H2+L+L2:N13				DESCRIPTORS			SEVERITY SCORE		
Your compound Number or	SMILES	STRUCTURES	24 h	48 h	3d	24 h	48 h	3d	24w coordinate	4 h	8 h	24 h	4 h	8 h	24 h
1	CCCCNC(=O) C1=CC2=[N+] (O)ON=C2C=	#¿NOMBRE?		R, Dark, S	R, Dark, S	0	3	3							
110	O=C1N(CCC2 =CC=CC=C2)[S](=O)(=O)N(	#¿NOMBRE?		T		0	0	0							
114	O=C1N(CC2= CC=CC=C2)[S] (=O)(=O)N(C	#¿NOMBRE?		R, Dark, S	R, Deg	0	3	4							
115	O=C1N(CCC2 =CC=CC=C2)[S](=O)(=O)N(	#¿NOMBRE?	R, S	R, Dark, S	R, Deg, S	2	3	4							
144	ClC1=CC=C(C =C1)C2=CSC(=NN=CC=CC3	#¿NOMBRE?	Red, O	R,Dark,Red, ppt	R, Deg	1	2	4	B6	red			0	0	0
187	CC1=CC=C(C= C1)C2=NSC(= N2)[S](=O)(=	#¿NOMBRE?	R, Deg, S	**D**	**D**	4	4	4	C5			S+, on sides	0	0	2
303	[O-][N+]1=C2C= CC(=CC2=[N+	#¿NOMBRE?		R, Deg, S	R, Deg, S	0	4	4	B5				0	0	0
328	CCCCNC(=O) NN=CC1=CC2 =[N+](O)C(=C	#¿NOMBRE?		Dark, ppt		0	1	0							
490	CC1=C(C(=O) NC2=CC=CC= C2)[N+](=C3C	#¿NOMBRE?	ppt	ppt		0	0	0							
898	C[S](=O)(=O) C1=CC=C(C=C C(O)C2=CC=C	#¿NOMBRE?	R, Dark, O	R, Deg	**D**	3	4	4	D5 (?)				0	0	0
1148	CCCCN1C(=O)C(=CC2=CC= C(S2)[N](=O)	#¿NOMBRE?		R		0	1	0							
1254	OC1=C(C=CC 2=CC=CC=C1 2)C(=O)C=CC	#¿NOMBRE?		Dark, S		0	2	0							
1267	O([H])C2=CC =C1C=CC=CC 1=C2C(=O)C=	#¿NOMBRE?	S	R, Deg	R, Deg, S	1	4	4	A6				0	0	0
1270	O([H])C2=CC =C1C=CC=CC 1=C2C(=O)C=	#¿NOMBRE?		Dark		0	1	0							

Table 3S. Clinic severity score used in the in vivo assay. Infected mice with F. hepatica.[58]

Ascites		Spleen		Number of lesions/hepatic lobe		Liver lobes	
**Score**	**Description**	**Score**	**Size**	**Score**	**Description**	**Score**	**Description**
0	None (normal cell content)	0	Normal	0	None	0	Healthy
1	Mild (medium cell content)	1	Splenomegaly (<2x)	1	<3 lesions	1	1hepatic lobe affected
2	Moderate (high cell content)	2	Splenomegaly (>2x)	2	>3 lesions	2	>2 hepatic lobes affected
3	Severe (high cell and blood content)			3	Complete affection of lobes		

Maximal score is 10.

Figure 2S. *in silico* exploration of the pharmacokinetic properties

TCZ

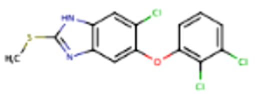

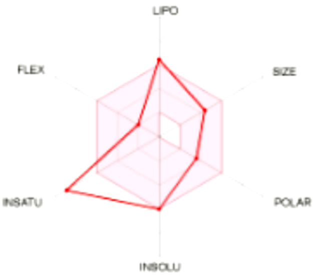

SMILES CSc1[nH]c2c(n1)cc(c(c2)Cl)Oc1cccc(c1Cl)Cl

Physicochemical Properties	
Formula	C14H9Cl3N2OS
Molecular weight	359.66 g/mol
Num. heavy atoms	21
Num. arom. heavy atoms	15
Fraction Csp3	0.07
Num. rotatable bonds	3
Num. H-bond acceptors	2
Num. H-bond donors	1
Molar Refractivity	89.36
TPSA	63.21 Å²

Lipophilicity	
Log $P_{o/w}$ (iLOGP)	3.00
Log $P_{o/w}$ (XLOGP3)	5.74
Log $P_{o/w}$ (WLOGP)	6.04
Log $P_{o/w}$ (MLOGP)	4.28
Log $P_{o/w}$ (SILICOS-IT)	5.52
Consensus Log $P_{o/w}$	4.92

Water Solubility	
Log *S* (ESOL)	-6.02
Solubility	3.46e-04 mg/ml ; 9.62e-07 mol/l
Class	Poorly soluble
Log *S* (Ali)	-6.84
Solubility	5.26e-05 mg/ml ; 1.46e-07 mol/l
Class	Poorly soluble
Log *S* (SILICOS-IT)	-7.47
Solubility	1.22e-05 mg/ml ; 3.40e-08 mol/l
Class	Poorly soluble

Pharmacokinetics	
GI absorption	High
BBB permeant	No
P-gp substrate	No
CYP1A2 inhibitor	No
CYP2C19 inhibitor	Yes
CYP2C9 inhibitor	Yes
CYP2D6 inhibitor	No
CYP3A4 inhibitor	Yes
Log K_p (skin permeation)	-4.42 cm/s

Druglikeness	
Lipinski	Yes; 1 violation: MLOGP>4.15
Ghose	No; 1 violation: WLOGP>5.6
Veber	Yes
Egan	No; 1 violation: WLOGP>5.88
Muegge	No; 1 violation: XLOGP3>5
Bioavailability Score	0.55

Medicinal Chemistry	
PAINS	0 alert
Brenk	0 alert
Leadlikeness	No; 2 violations: MW>350, XLOGP3>3.5
Synthetic accessibility	2.36

187

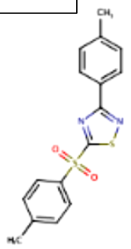

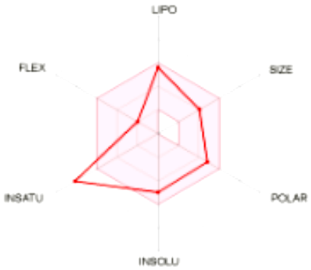

SMILES Cc1ccc(cc1)c1nsc(n1)S(=O)(=O)c1ccc(cc1)C

Physicochemical Properties	
Formula	C16H14N2O2S2
Molecular weight	330.42 g/mol
Num. heavy atoms	22
Num. arom. heavy atoms	17
Fraction Csp3	0.12
Num. rotatable bonds	3
Num. H-bond acceptors	4
Num. H-bond donors	0
Molar Refractivity	87.21
TPSA	96.54 Å²

Lipophilicity	
Log $P_{o/w}$ (iLOGP)	2.92
Log $P_{o/w}$ (XLOGP3)	4.28
Log $P_{o/w}$ (WLOGP)	4.74
Log $P_{o/w}$ (MLOGP)	2.47
Log $P_{o/w}$ (SILICOS-IT)	4.42
Consensus Log $P_{o/w}$	3.76

Water Solubility	
Log *S* (ESOL)	-4.96
Solubility	3.63e-03 mg/ml ; 1.10e-05 mol/l
Class	Moderately soluble
Log *S* (Ali)	-6.02
Solubility	3.16e-04 mg/ml ; 9.55e-07 mol/l
Class	Poorly soluble
Log *S* (SILICOS-IT)	-6.63
Solubility	7.78e-05 mg/ml ; 2.36e-07 mol/l
Class	Poorly soluble

Pharmacokinetics	
GI absorption	High
BBB permeant	No
P-gp substrate	No
CYP1A2 inhibitor	Yes
CYP2C19 inhibitor	Yes
CYP2C9 inhibitor	Yes
CYP2D6 inhibitor	No
CYP3A4 inhibitor	Yes
Log K_p (skin permeation)	-5.28 cm/s

Druglikeness	
Lipinski	Yes; 0 violation
Ghose	Yes
Veber	Yes
Egan	Yes
Muegge	Yes
Bioavailability Score	0.55

Medicinal Chemistry	
PAINS	0 alert
Brenk	0 alert
Leadlikeness	No; 1 violation: XLOGP3>3.5
Synthetic accessibility	3.23
